# Increasing typhoon impact and economic losses due to anthropogenic warming in Southeast China

**DOI:** 10.1038/s41598-022-17323-8

**Published:** 2022-09-08

**Authors:** Mingfeng Huang, Qing Wang, Maofeng Liu, Ning Lin, Yifan Wang, Renzhi Jing, Jianping Sun, Hiroyuki Murakami, Wenjuan Lou

**Affiliations:** 1 Institute of Structural Engineering, College of Civil Engineering and Architecture, Zhejiang University, Hangzhou, 310058 China; 2 Shanghai Institute for Advanced Study, Zhejiang University, Shanghai, 201203 China; 3 Rosenstiel School of Marine and Atmospheric Science, University of Miami, Miami, USA; 4 Department of Civil and Environmental Engineering, Princeton University, Princeton, NJ USA; 5 NOAA/Geophysical Fluid Dynamics Laboratory, and Atmospheric and Oceanic Sciences Program, Princeton University, Princeton, NJ USA

**Keywords:** Atmospheric dynamics, Natural hazards

## Abstract

Despite a variety of studies on the tropical cyclone (TC) response to climate change, few of them have examined the projected damages of future TCs. Here we quantify the impact of anthropogenic warming on TC-induced damages in the late twenty-first century along the coasts of Southeast China based on convection-permitting TC simulations and machine-learning-based damage models. We found that if the area’s 10 super typhoons between 2013 and 2019 were to occur at the end of the century under the high emissions RCP8.5 scenario, they would have on average a 12% ± 4% increase in landfall intensity, 25% ± 23% increase in precipitation, and 128% ± 70% increase in economic losses, compared to historical simulations. We also found a significant increase in the full risk profile. The estimated typhoon loss with a 50-year return period for Zhejiang, Fujian, Guangdong, and Hainan (four most typhoon-prone provinces among the seven provinces in the region) would increase by 71%, 170%, 20%, and 85%, respectively, towards the end of the century even under the lower emissions RCP4.5 pathway. Our findings imply the need to design effective local hazard mitigation measures to reduce future typhoon risks.

## Introduction

The Western North Pacific Ocean (WNP) is the most active tropical cyclone (TC) basin, accounting for almost one-third of the world’s annual TCs. Severe TCs, also called typhoons (with maximum 10-m wind speed at least 32.7 m/s) in WNP, cause devastating losses of life and property in Southeast China, where both the population and economy are growing rapidly. One of the most prominent examples is Typhoon Lekima (2019), which made landfall in mainland China with a major hurricane intensity and caused 56 fatalities and a direct economic loss of 53.72 billion CNY (http://www.ndrcc.org.cn/). Lekima produced the record of daily rainfall accumulation in both Zhejiang and Shandong Province since the weather observation system in China was first established in 1949, with the resultant flooding a primary cause of fatalities and property damages. Besides Lekima, the recent high typhoon activity in WNP has posed a serious threat to Southeast China. For instance, there were 16, 10, and 9 super typhoons (maximum 10-m wind speed > 43.6 m/s, equivalent to at least Category 2 hurricanes) in the 2015, 2016, and 2018 WNP typhoon seasons, respectively, in comparison to 8 on average over 1948–2017.

A number of studies have been devoted to the long-term trend in TC activity in the WNP^1–3^. The intensity of typhoons striking East and Southeast Asia during 1977–2014 was enhanced by 12–15%, and the proportion of Category 4 and 5 storms doubled or even tripled^1^. A recent study found that the distance to land of TCs lifetime maximum intensity has decreased by about 30 km per decade globally over the period of 1982–2018^2^. Also, an increasing trend of lifetime maximum intensity of rapidly intensifying TCs was witnessed over the past few decades for the coastal regions in the WNP basin^3^. These observed trends have been tied to internal climate modes, aerosol change, and global warming^2–7^. Looking into the future, although the influence of anthropogenic forcing on some aspects of TC activity remains elusive^4–7^, there is increased confidence that the number and proportion of intense TCs will increase under a warming climate^8–11^. In addition, climate modeling studies project increased rainfall rates associated with TCs under a variety of warming scenarios^12,13^. The projected increase in TC intensity and storm rainfall rate poses an increasing threat to life and property in coastal regions. TC-induced storm surge and sea level rise (SLR) are also important factors for increased coastal flood hazards^14,15^.

With the advances of dynamical modelling of TCs, changes in TC damages can be assessed by combining atmospheric science and economics^16^, given various emission scenarios^17^ and socioeconomic development pathways^18^. For example, a climate change signal has been identified in historical U.S. hurricane losses from an economic point of view by a regression-based approach^19^. A statistical relationship linking the maximum 10-m wind speed of hurricanes and the economic losses was developed for the U.S. Atlantic and Gulf Coasts and used to assess the influence of climate change on TC damages with the aid of a synthetic TC model^20^. The Community Earth System Model was combined with the TC damage model to estimate future changes in TC-induced damages on the global scale^21^. The study found that the global TC damage is mostly dominated by East Asia due to a large number of strong Pacific storms^1^, which indicates that the regional impact studies are urgently needed^22^ to guide policy makers to take appropriate risk-reduction actions and hence limit the impact of climate change.

This study aims at advancing our understanding of anthropogenic effects on regional TC impacts by quantifying both the “worst-case-scenario” damages and full economic risk profiles in coastal areas of Southeast China through the integration of coupled atmosphere–ocean general circulation models (CGCMs^23,24^), the mesoscale Weather Research and Forecasting (WRF) model^25,26^, and machine-learning-based damage models. The damage models on a regional level are developed based on the deep neural network (DNN) to establish links between typhoon damages in Southeast China and six meteorological variables including storm intensity (i.e., minimum sea-level pressure (SLP) and maximum 10-m wind speed), storm size (i.e., storm radius of winds greater than 34 knots), daily site-maximum precipitation, daily area-mean precipitation, and astronomical high tide using historical data and are employed for estimating future socioeconomic typhoon impacts on Southeast China (see “Methods”).

We conduct convection-permitting WRF control simulations for a collection of 10 historical super typhoons that produced devastating damages in Southeast China (referred to as SuperTYs hereafter). We also simulate pre-industrial and future episodes of the same 10 storms by placing them in the climate conditions of 1860–1880 or at the end of the twenty-first century under the RCP4.5 and RCP8.5 scenarios. This analysis is achieved through the so-called pseudo-global warming (PGW) approach^27,28^, in which initial and boundary conditions for the control simulations are modified with pre-industrial perturbations or future projections derived from the Geophysical Fluid Dynamics Laboratory (GFDL) High-Resolution Forecast-Oriented Low Ocean Resolution (HiFLOR^23,24^) experiments or the Coupled Model Intercomparison Project Phase 5 (CMIP5) models. Changes in storm activity including intensity and rainfall for the 10 SuperTYs are further used to drive the DNN models to investigate how the damages from the SuperTYs would respond to climate warming. Because HiFLOR shows good skill in simulating the intensity spectrum of TCs including Categories 3–5 TCs^23,24^, we also couple the landfalling typhoon samples directly simulated from a 70-year HiFLOR experiment^24^ under the RCP4.5 scenario with the DNN damage models to estimate the full hazard risk profiles in Southeast China by the end of the twenty-first century.

### Typhoon modeling

As a module of the WRF system, the Advanced Hurricane Weather Research and Forecasting (AHW) model^25,26^ was developed especially for the research on typhoon/hurricane simulation and real-time prediction. Based on the AHW model (Version 3.7), the convection process of the inner typhoon center is explicitly resolved with high-efficiency movable 4-km grids while the interaction between the atmospheric environment and the typhoon circulation is also considered in the outer large computational domain. We perform the 10-member ensemble simulation using the stochastic kinetic energy backscatter scheme (SKEBS)^29^, which captures the uncertainties in unresolved convection scales by introducing temporally and spatially correlated perturbations to the rotational wind components and potential temperature.

The control simulations consisted of hindcasts of the 10 SuperTYs in the historical conditions in which they actually occurred. We also performed the simulations of the 10 SuperTYs under the pre-industrial and future climate scenarios via the PGW approach^27,28^. The PGW simulations were conducted by adding perturbations to the initial and boundary conditions for historical control simulations. For pre-industrial typhoon simulations, the climate perturbations were computed as the differences between 1860 and 1880 monthly climatology (pre-industrial climate) and 2000–2020 monthly climatology (historical climate) from general circulation models (GCMs). For future climate simulations, the climate differences between 2080 and 2100 monthly climatology (future climate) and 2000–2020 monthly climatology were used as perturbations. The climate perturbations from two individual GCM models (FGOALS-s2 and CCSM4), 10-GCM model ensemble mean from the CMIP5 project, and HiFLOR^23,24^ were used in the future typhoon PGW simulation under low (RCP 4.5) or high (RCP 8.5) emission scenarios (see more details in “Methods”).

HiFLOR has a horizontal resolution of approximately 25 km for the atmosphere and land components and a 1° resolution for sea ice components, and it is capable of generating intense storms^24^. In a previous study, 70-year control simulations of HiFLOR experiments were performed by nudging the model’s sea surface temperatures (SSTs) towards the climatological SST over the period 1986–2005^23^. Future “early” and “late” climate HiFLOR experiments were conducted to project the storm climatology during 2016–2035 and 2080–2100, respectively, with the climatological SST derived from a multi-model mean of 17 CMIP5 models for the RCP4.5 pathway^24^. Here we focus on the “late” (2081–2100) HiFLOR experiments because their differences are more significant to reflect the climate change response of TCs in HiFLOR. For scenario analysis, we used the “late” HiFLOR climate conditions to drive the AHW model for the simulations of the 10 SuperTYs in the future climate condition. To estimate future typhoon damage risks in Southeast China during the late twenty-first century, we identified landfalling typhoon events in Southeast China from the HiFLOR “late” experiments. Typhoon meteorological variables of the identified typhoon events served as direct inputs to estimate typhoon damages by the developed DNN typhoon damage models (see “Methods”).

## Results

**Figure 1:**
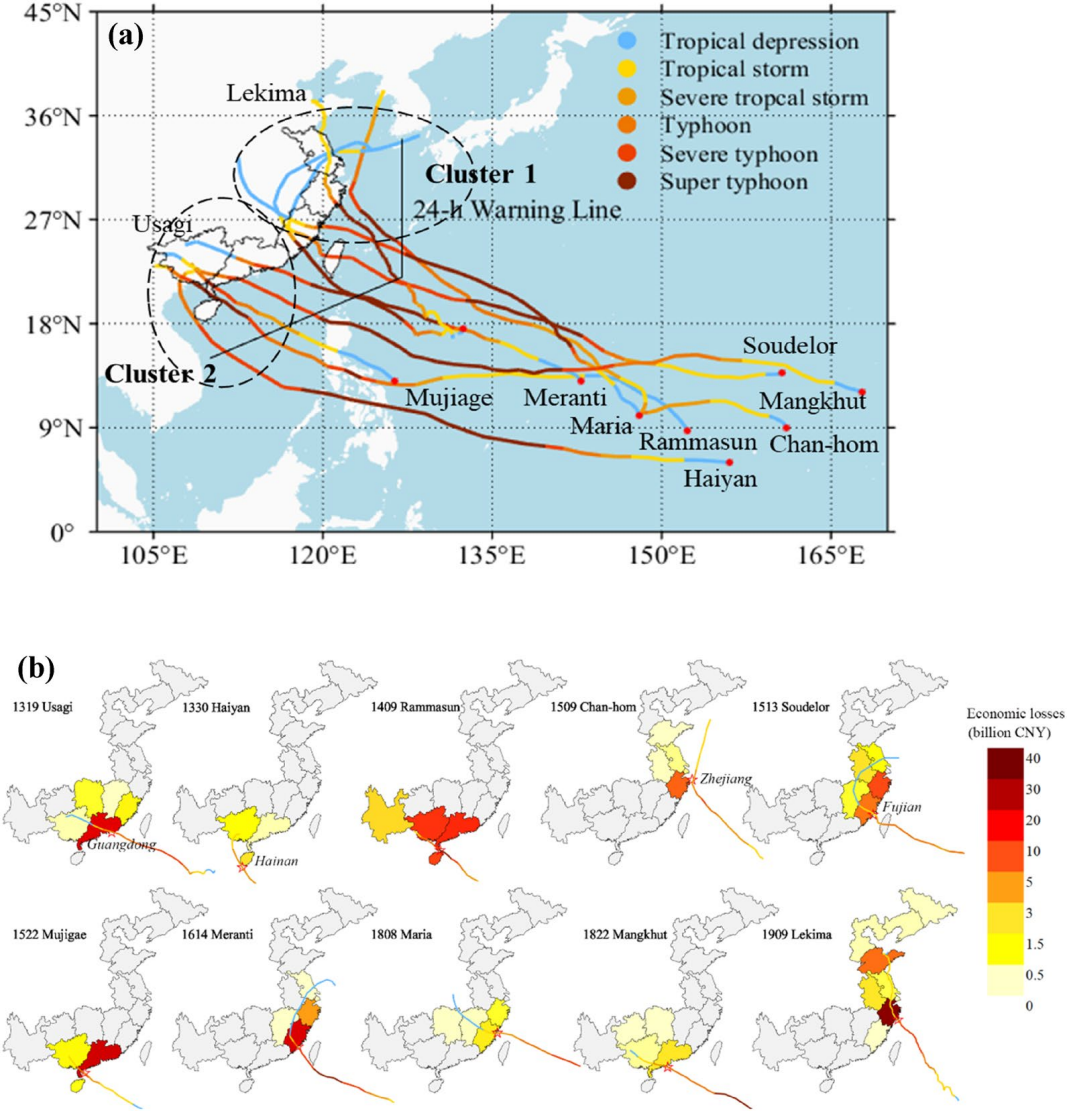
Ten historical super typhoons and damage in coastal area of China. (**a**) Tracks of the 10 landing SuperTYs. (**b**) Direct economic loss (consumer price index CPI-2013 adjusted losses) distribution. Shadings indicate the damage level of TC-affected provinces. Lines indicate the tracks of the selected typhoons. Historical damage data are mainly from *China Meteorological Yearbook*. The four directly hit provinces, i.e., Guangdong, Hainan, Zhejiang and Fujian, are labeled. Maps were generated by MATLAB R2021a (http://www.mathworks.com/).

The 10 historical storms selected over 2013–2019 are landfalling SuperTYs with a lifetime maximum 10-m wind speed no less than 51 m/s, approximately equivalent to major hurricanes. The 10 SuperTYs (Fig. 1a) represent well the most catastrophic typhoons in Southeast China (Fig. 1b). The tracks of the 10 SuperTYs fall into Cluster 1 or Cluster 2 groups identified by a clustering analysis^1^ (Fig. 1a) that classifies the typhoons of the WNP basin into four distinct regional groups in terms of their geographic locations of genesis and tracks. Cluster 1 and 2 groups include more than 60% of typhoons over the entire WNP with a high landfall rate (~ 85%)^1^, with the most pronounced historical increasing trends of intensity and intensification rate^2^. Cluster 1 typhoons, namely, Chan-hom, Soudelor, Meranti, Maria, and Lekima, originated from east of the Philippines, then traveled northwestwards-to-northwards, and made landfall over East Asia. Cluster 2 typhoons, namely, Usagi, Haiyan, Rammasun, Mujigae, and Mangkhut, formed slightly westward of those in Cluster 1, over the South China Sea, moved more directly towards the west-to-northwest, and struck Southeast Asia (including South China, the Philippines, and Vietnam). The landfall details of the 10 SuperTYs, i.e., landfalling locations and affected provinces of Chinese coast, are given in Supplementary Table 1.

The direct economic losses for the 10 SuperTYs are presented in Fig. 1b (see Supplementary Table 2 for more details). The storm impacts mainly covered seven coastal provinces, namely, from north to south, Shandong, Jiangsu, Zhejiang, Fujian, Guangdong, Guangxi, and Hainan Provinces of Southeast China. Among these provinces, Zhejiang, Fujian, Guangdong, and Hainan are the most typhoon-prone regions with the largest average annual typhoon losses in China.

**Figure 2:**
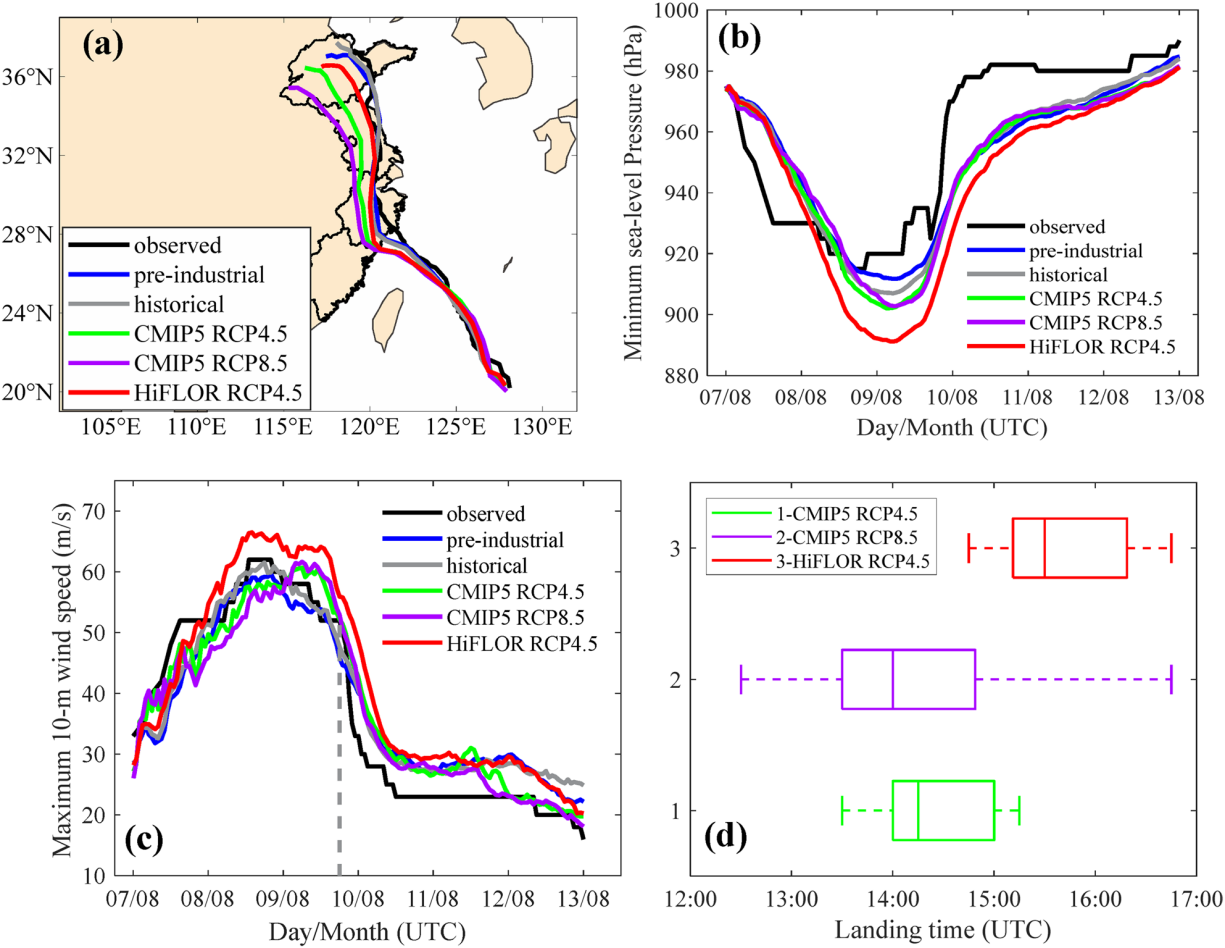
Tracks, time series of minimum SLP, time series of maximum 10-m wind speed, and landing time for Typhoon Lekima. (**a**–**c**) Observations (black), ensemble mean of the pre-industrial (blue), historical (gray), RCP4.5 of HiFLOR (red), RCP4.5 (green), and 8.5 (purple) of CMIP5 mean simulations at 4-km resolution for Typhoon Lekima. (**a**) Tracks. (**b**) The time series of minimum SLP (hPa). (**c**) Maximum 10-m wind speed. Observed landfall time is marked with a vertical gray dashed line. **(d)** TC landing time in boxplot for the ensemble simulation under three future PGW conditions**.** Maps were generatedbyMATLABR2021a(http://www.mathworks.com/).

Figure 2 shows the WRF-based hindcast (control) simulation of SuperTY Lekima. The hindcasted typhoon track matches well with the historical record (Fig. 2a). The time series of minimum SLP and maximum 10-m wind speed (Fig. 2b,c) show that the hindcasted intensity is relatively close to the observation. Similar to the data for Lekima, the hindcasted historical tracks and intensities of all 10 SuperTYs agree well with observations (see Table 1 and Supplementary Fig. 1), except that the maximum 10-m wind speeds are underestimated for the early simulation stage of SuperTYs Haiyan, Maria, and Mangkhut (Supplementary Fig. 2d,p,r). Notably, all 10 SuperTYs underwent rapid intensification. A failure to reproduce rapid intensification of intense TCs was reported in the recent convection-permitting regional climate model simulations^28^. Our hindcast run shows skill in simulating the rapid intensification period of Lekima with an observed intensification rate of 22 m/s in 24 h. The rapid intensification stages that occurred right before landfall are captured well by the WRF hindcast simulations (21 m/s in 24 h) for Lekima (historical simulation in Fig. 2c), Rammasun, Chan-hom, Mujigae, and Meranti (Supplementary Fig. 2f,h,l,n). The other five SuperTYs underwent rapid intensifications in the open ocean far away from the shore, beyond the simulation periods.

**Table 1:** Maximum 10-m wind speed at landfall (m/s).

Typhoon (year)	Observed	Historical hindcasts (mean ± $\sigma )$	CMIP5 RCP4.5 minus Historical	CMIP5 RCP8.5 minus Historical	HIFLOR RCP4.5 minus Historical
Usagi (2013)	50.0	47.9 ± 1.3	0.1 (0%)	2.3 (5%)	1.9 (4%)
Haiyan (2013)	40.0	40.4 ± 2.0	3.0 (7%)	4.9 (12%)	3.5 (9%)
Rammasun (2014)	60.0	63.2 ± 4.3	3.9 (6%)	8.2 (13%)	5.1 (8%)
Chan-hom (2015)	42.0	37.0 ± 2.2	2.3 (6%)	4.1 (11%)	2.2 (6%)
Soudelor (2015)	38.0	39.4 ± 2.4	3.6 (9%)	6.3 (16%)	3.3 (8%)
Mujigae (2015)	52.0	53.1 ± 2.4	1.5 (3%)	9.0 (17%)	4.1 (8%)
Meranti (2016)	52.0	49.7 ± 2.5	4.6 (9%)	6.2 (13%)	5.5 (11%)
Maria (2018)	42.0	44.5 ± 1.6	− 0.7 (2%)	2.7 (6%)	2.6 (6%)
Mangkut (2018)	48.0	46.7 ± 1.9	1.2 (3%)	6 (13%)	4.7 (10%)
Lekima (2019)	52.0	51.0 ± 1.3	5.8 (11%)	7.3 (14%)	9.1 (18%)

*The ensemble-mean difference in typhoon maximum 10-m wind speed (m/s) is given. Maximum wind speed is estimated by 10-member ensemble simulations. The change percentage of the ensemble-mean wind speed compared to hindcasts under the historical conditions is given in brackets.

The skill of WRF in reproducing track and intensity of the 10 SuperTYs gives us confidence to further explore these storms by the PGW approach under pre-industrial or projected future climate states driven by the mean of 10 CMIP5 models (Supplementary Table 3) for both RCP4.5 and RCP8.5 scenarios. Another PGW-based simulation was conducted using the future climate states derived from the HiFLOR model with a high spatial resolution of approximately 25 km under the RCP4.5 scenario^24^. The maximum 10-m wind speed and minimum SLP time series are indistinguishable between pre-industrial and historical simulations for Lekima (Fig. 2b,c) and other typhoon events (Supplementary Fig. 2). All 10 typhoon events exhibit higher intensities at their landfalls under the three future climate conditions, compared to the corresponding historical ones (Table 1). The results suggest a significant increase (12% ± 4%, i.e., the average and the standard deviation of the maximum 10-m wind speed) in landfall intensity by the end of the century if these typhoons were to occur in future warmer climates under RCP8.5. Although relatively noticeable differences exist between modeled and observed maximum 10-m wind speed (Table 1), such differences are insignificant, as indicated by statistical tests with a 95% confidence interval.

The maximum of landfall intensity increase (18%) compared to the historical record is observed for Typhoon Lekima under the HiFLOR RCP4.5 climate. We note that for Lekima, the future storm under the HiFLOR RCP4.5 experiment intensified more than those under the CMIP5 RCP4.5 and RCP8.5 experiments. The greater intensification of Lekima under HiFLOR RCP4.5 is also shown by the greater decrease in minimum SLP at landfall (see Supplementary Table 4). This greater intensification in HiFLOR RCP4.5 cannot be attributed to the increments of SST between future and historical climate (as shown in Supplementary Figs. 3 to 4), as larger increments of monthly mean SSTs are observed for CMIP5 RCP8.5 than for HiFLOR RCP4.5. As shown in Fig. 2d, the mean landfall time of the simulated typhoon under HiFLOR RCP4.5 is delayed among the 10 ensemble members compared to the other two future PGW cases with the same initial time of simulation. The longer duration of simulated typhoons over the ocean allows more time for storm intensification, which partially explains the unexpected stronger storm intensity resulting from the HiFLOR RCP4.5 simulation. The simulated storm tracks are generally not sensitive to anthropogenic perturbations derived from either the CMIP5 models or HiFLOR (Fig. 2a and Supplementary Fig. 1), although the projected TC tracks tend to shift southwestward after landfall, probably due to enhanced easterlies in Southeast China^30^ in response to climate warming.

To further understand the uncertainty due to inter-model spread in CMIP5, we perform PGW-based simulations of Lekima, Haiyan, and Soudelor using individual model climate perturbation results under future (2080–2100) climate scenarios (see Supplementary Figs. 5 to 10). Lekima, Haiyan, and Soudelor all show an intensification in the future warmer climate with some spread in terms of different CMIP5 models. These results confirm that the model-to-model difference has some impact on PGW simulations. For example, significantly lower minimum sea-level pressures are observed for future Lekima, Haiyan, and Soudelor under RCP8.5 than those under RCP4.5 based on the FGOALS-s2 model. The two concentration pathways do not make any noticeable difference in minimum SLPs in the PGW simulation of the three super typhoons based on the CCSM4 model. These different intensity responses may be related to the slightly different tracks simulated in the CMIP5 models. The projected TC track of Haiyan shifts southwestward (by 1.7°) for mean CMIP5 RCP8.5 compared to the observed path (Supplementary Fig. 1b). When FGOALS-s2 or CCSM4 are used, the future Haiyan track for RCP8.5 shifts northeastward (by 1.4°) or shows little change compared to the observation (as shown in Supplementary Fig. 7b,c). Generally speaking, a “fair” comparison of intensity responses to climate perturbations may be made as long as the track deviation is within the criterion of about 3°^28^.

Next we analyze anthropogenic changes in rainfall in terms of site-maximum daily precipitation and daily area-mean precipitation at landfall, which are important metrics contributing to typhoon-induced damages. The maximum daily precipitation was recorded at observational sites of the China Meteorological Administration (CMA) for historical typhoons, and the daily area-mean precipitation is calculated by averaging the daily precipitation from the meteorological stations located in the same province. To match with each observational site, the daily site-maximum and area-mean precipitation from the WRF simulations are evaluated over a square region of 25 km × 25 km centered on the corresponding observational site. The WRF model is capable of capturing the timing and magnitude of maximum precipitation at the observational sites (Supplementary Fig. 11). However, a few historical simulation results of precipitation do not compare well with the observation due to the shifted storm tracks of the simulation from those in the observation. For Typhoon Lekima, the WRF model produces rainfall over a more extensive area than in the CMA observation (Supplementary Fig. 12), especially for the landfalling period. As shown in Fig. 2a, the simulated track of Lekima is slightly south of the observed one such that severe convection of landfalling Lekima might be triggered over Zhejiang’s southeastern mountains. The complex terrain-induced severe convection may be the primary reason for rainfall overestimation and larger rainfall coverage around 28°N. On the other hand, the simulated maximum daily precipitation of Typhoon Chan-hom of 144.7 mm is well below the observed value of 267.7 mm. The underestimation of site rainfall is expected since the simulated Chan-hom track is farther away from the mainland than the observation, as shown in Supplementary Fig. 1d. For future simulations, we find that anthropogenic warming significantly enhances rainfall at landfall for the 10 SuperTYs (see Table 2 and Supplementary Table 5). On average, the increase of maximum daily precipitation at landfall reaches 18% ± 17%, 15% ± 18%, and 25% ± 23%, corresponding to future climate scenarios of HiFLOR RCP4.5, CMIP5 RCP4.5, and CMIP5 RCP8.5, respectively.

**Table 2:** Maximum daily precipitation at landfall (mm).

Typhoon (year)	Observed	Historical hindcasts (mean ± $\sigma )$	CMIP5 RCP4.5 minus Historical	CMIP5 RCP8.5 minus Historical	HiFLOR RCP4.5 minus Historical
Usagi (2013)	143.0	195.4 ± 22.9	60.6 (31%)	32.1 (16%)	− 12.1(6%)
Haiyan (2013)	320.4	314.0 ± 82.3	169.3(54%)	249.1 (79%)	181.3 (58%)
Rammasun (2014)	186.3	303.7 ± 58.9	14.0 (5%)	96.2 (32%)	70.4 (23%)
Chan-hom (2015)	267.7	144.7 ± 49.4	1.2 (1%)	− 5.2 (4%)	27.2 (19%)
Soudelor (2015)	244.4	500.1 ± 44.6	151.0 (30%)	227.8 (46%)	94.8 (19%)
Mujigae (2015)	219.0	305.8 ± 43.2	19.8(6%)	69.8 (23%)	16.2 (5%)
Meranti (2016)	172.7	395.6 ± 39.0	10.3 (3%)	73.0(15%)	36.5 (9%)
Maria (2018)	141.0	113.8 ± 21.8	− 0.6(1%)	16.5 (14%)	18.4 (16%)
Mangkut (2018)	173.5	245.7 ± 30.0	13.9 (6%)	46.3 (19%)	31.7 (13%)
Lekima (2019)	291.0	325.5 ± 28.8	56.4 (17%)	47.0 (14%)	69.3 (21%)

**Figure 3:**
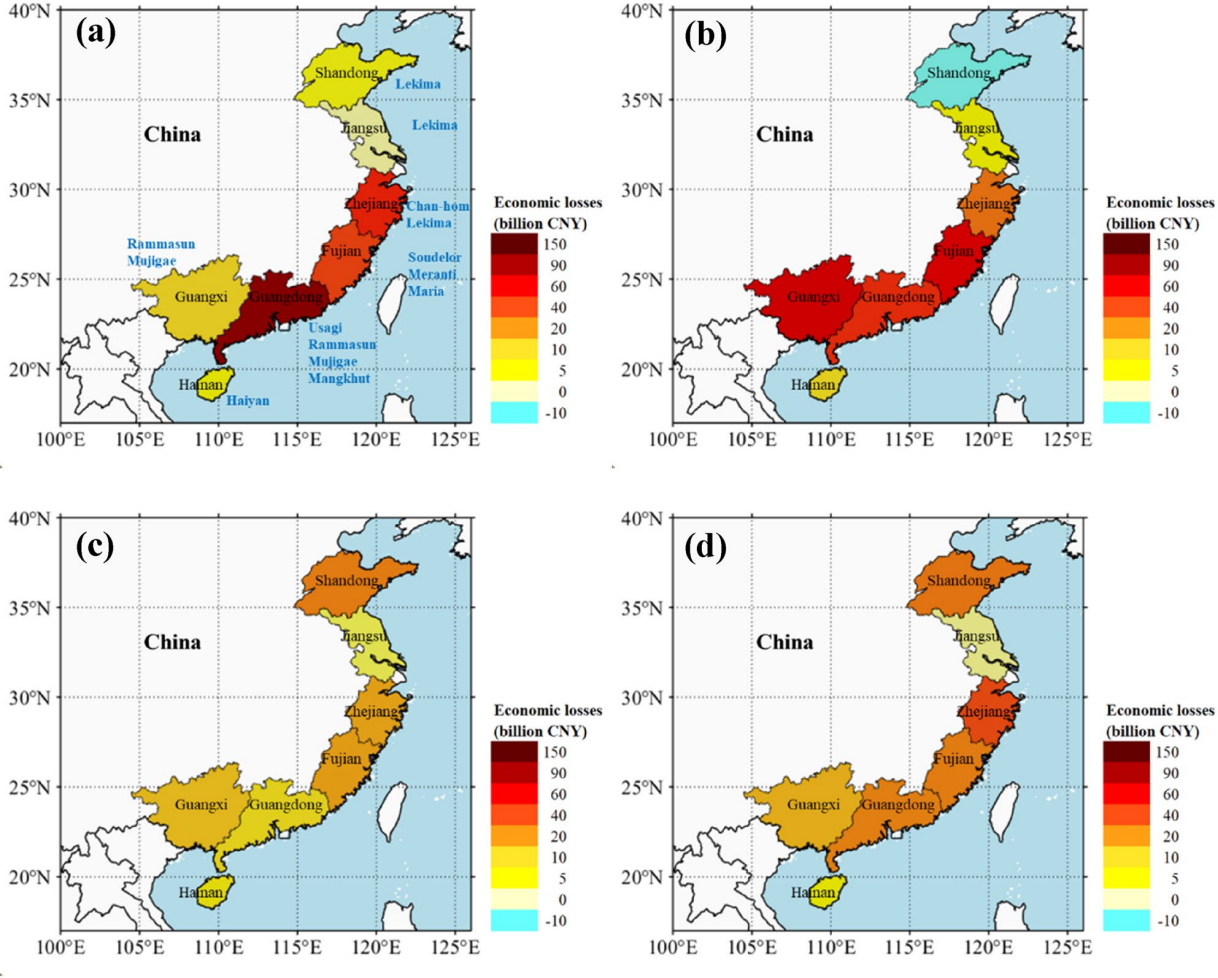
Estimated direct economic loss (CPI-2013 adjusted losses) of the 10 SuperTYs in historical and future climates by DNN models. **(a)** Direct loss estimation from historical simulation of the 10 SuperTYs. **(b)** Projected change of direct economic loss for CMIP5 mean RCP8.5 relative to historical estimation. **(c)** Projected change of direct economic loss for CMIP5 mean RCP4.5 relative to historical estimation. **(d)** Projected change of direct economic loss for HiFLOR RCP4.5 relative to historical estimation. Maps were generated by MATLAB R2021a (http://www.mathworks.com/).

To assess future typhoon impacts in Southeast China, the DNN models were designed and trained using six typhoon meteorological variables and the collected province-level economic loss data. Since ensembles of neural networks are known to be much more robust than individual networks, 10 neutral network models were independently developed to form a model ensemble for predicting typhoon damage of each province. Figure 3 presents the direct economic losses of the 10 SuperTYs estimated by the DNN model ensembles under both historical and future climate scenarios. The observed and the DNN-estimated historical losses show similar spatial distribution patterns in the seven coastal provinces (see Fig. 3a and Supplementary Fig. 13), indicating the good performance of the established province-level DNN damage model ensembles (see also comparison of simulation and observation in Supplementary Fig. 14). Building on the skill of DNN damage model in reproducing historical damages, it was further applied to predict the future economic losses for all 10 SuperTYs under climate conditions of CMIP5 mean RCP 8.5, CMIP5 mean RCP4.5, and HiFLOR RCP4.5. The 10 SuperTYs were found to have a 128% ± 70% increase of estimated economic losses in CMIP5 mean RCP8.5 compared to historical simulations, corresponding to an increase in total losses in the Chinese coastal provinces from 218 billion CNY to about 442 billion CNY. Except for Shangdong and Zhejiang provinces, the projected increases of direct economic loss for the other five coastal provinces in CMIP5 mean RCP8.5 (see Fig. 3b) are noticeably larger than the corresponding losses under CMIP5 mean RCP4.5 (see Fig. 3c) and HiFLOR RCP4.5 (see Fig. 3d). For example, Guangdong would suffer a maximum loss of 155 billion CNY in CMIP5 mean RCP8.5 among the seven coastal provinces in Southeast China (see Supplementary Fig. 14), which is 33% and 17% larger than the corresponding losses under CMIP5 mean RCP4.5 and HiFLOR RCP4.5, respectively. For Zhejiang province, the future maximum economic loss (90 billion CNY) corresponds to the HiFLOR RCP4.5 climate (Fig. 3d), under which Lekima shows the greatest intensity among three future climate conditions (see Fig. 2c). The future loss of Shangdong under the RCP8.5 scenario is surprisingly smaller than the simulated historical loss (due to Lekima), as indicated in Fig. 3b with negative value (blue shading). This result might be explained by the much-skewed track of a future Lekima from the observed typhoon path. The future track towards deep inland reduces the intensity of Lekima affecting Shandong, compared to the historical hindcast when the storm center moved into Shangdong on 12 August, 2019 (See the tail stage of plots in Fig. 2c). Since Shangdong Province is affected only by Lekima among the 10 selected storms, the future typhoon loss of Shangdong (i.e., 1.6 billion CNY) due to a future Lekima under the high emission scenario becomes even smaller than that of the simulated historical Lekima (see Supplementary Fig. 14).

HiFLOR’s capability of generating the high-intensity TCs^24^ provides a good opportunity to estimate the full loss risk profile in Southeast China. The direct economic loss of each future TC event in the 70-year HiFLOR experiments could be predicted by the province-level DNN models using the meteorological variables associated with each identified HiFLOR landfall TC in the historical and RCP4.5 scenarios. However, recognizing that the biases in the HiFLOR simulation of TC frequency and TC intensity in Pacific Ocean are the common features of CGCMs that resolve the strongest TCs^24^, we first bias-correct the projected TC frequency and wind speeds from the “late” HiFLOR experiment (2081–2100) before using those TC meteorological variables to estimate the direct economic loss. TC frequency in the HiFLOR control simulation is bias-corrected against the CMA typhoon observations during the period 1986–2005 (see “Methods” and Supplementary Fig. 15). Assuming that the model biases can be inherited by the future projections, we estimate future storm frequency by frequency bias correction^31,32^ (see Supplementary Fig. 15). The maximum 10-m wind speeds are also bias-corrected against the CMA observations by using a quantile mapping bias-correction algorithm (see “Methods” and Supplementary Fig. 16).

**Figure 4:**
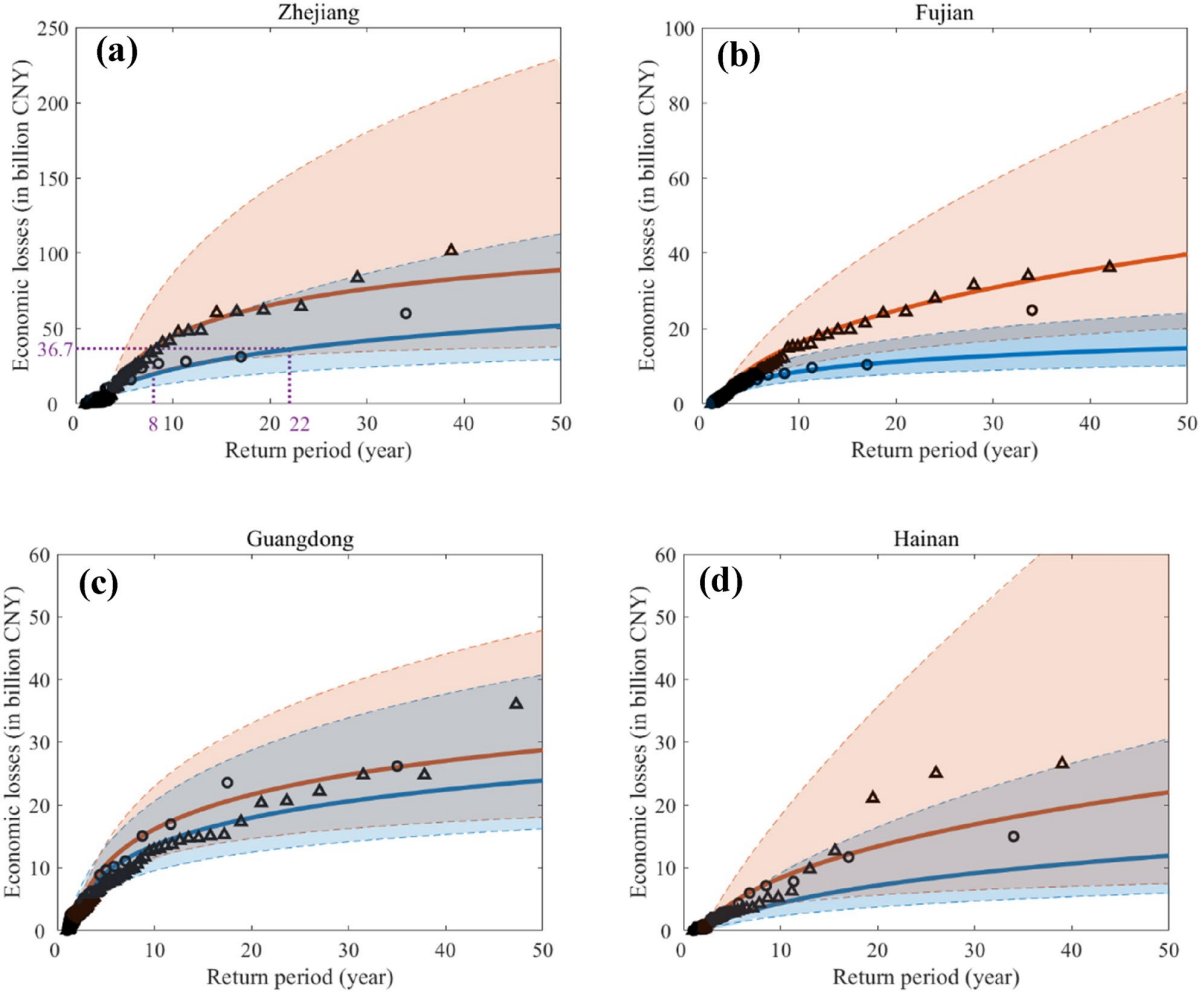
Return period curves of typhoon economic losses for four coastal provinces of China: Zhejiang (**a**), Fujian (**b**), Guangdong (**c**) and Hainan (**d**). Red lines correspond to the future climate during 2080 to 2100. Blue lines correspond to the historical period of 1980 to 2018. Solid lines represent the best estimates of economic loss return periods. Shaded areas cover the very likely range estimates (i.e., 95% statistical confidence interval). Black triangles represent predicted future typhoon-induced losses; black hollow circles represent historical observed typhoon losses. Maps were generated by MATLAB R2021a (http://www.mathworks.com/).

Figure 4 shows return period curves of both historical observed and future projected typhoon economic loss for the four typhoon-prone coastal provinces of Southeast China. The return periods of historical typhoon damage levels are estimated based on historical data (see “Methods”). For instance, estimated losses of Zhejiang, Fujian, Guangdong, and Hainan with a 50-year return period are 51.9, 14.7, 23.9, and 11.9 billion CNY, respectively (see the solid blue lines in Fig. 4). By the end of twenty-first century, the estimated typhoon loss risk (see the red lines in Fig. 4) shows a substantial increase due to TC climatology change even without considering SLR and future exposure growth of coastal population and wealth. For example, Zhejiang, Fujian, Guangdong, and Hainan Provinces would face a 71%, 170%, 20%, and 85% increase, respectively, of future typhoon economic loss at a 50-year return period, and the total damage increase for the four provinces reaches 75%.

Under both historical and future climates, Zhejiang Province exhibits the greatest typhoon-induced damages. Typhoon Lekima produced a loss of almost 36.7 billion CNY (CPI-2013 adjusted losses) to Zhejiang Province in 2019, translating to a return period of 22 years in the historical period (see Fig. 4a). According to the future return period of losses in Zhejiang, the same damage level of 36.7 billion CNY might occur on average every eight years (see Fig. 4a), much more frequently than in the historical period. Guangdong Province shows the lowest increase of potential typhoon damage in the late twenty-first century compared to Zhejiang, Fujian, and Hainan Provinces. The results indicate that Guangdong Province may be better prepared in terms of typhoon risk mitigation through 1980 to 2018 than the other three provinces. Since the province-level DNN models are trained independently using the data from each province, they implicitly account for the differences in hazard resilience of the provinces.

## Discussion

SuperTY Lekima in 2019 produced catastrophic damages in Zhejiang province. There is an urgent need to project future typhoon activity under climate change and assess its impact on Southeast China as the TC precipitations and the TC intensity are, with growing confidence, projected to increase under anthropogenic warming. Here, for the first time, we quantify the increase in typhoon damage at the province level by the end of twenty-first century for Southeast China due to TC climatology change.

The increase of typhoon damage risk under future climate could be attributed mainly to stronger storm intensity (5% and 12% increases on average under the RCP4.5 and RCP8.5 warming scenarios, respectively) and heavier precipitation (15% and 25% increases on average under the RCP4.5 and RCP8.5 warming scenarios, respectively), as evidenced in Tables 1 and 2. Here we focus on the effects of anthropogenic warming on typhoon impact by assuming the same exposure to historical typhoons and future typhoons for each province of concern. However, a slight difference in typhoon tracks could influence the actual loss greatly, and thus our results are subject to uncertainties as discussed. Nevertheless, the deviations of simulated tracks are within the previously considered deviation threshold of 3° of latitude or longitude that would enable a “fair” comparison of the TC responses to current and future climates^28^. In addition, our regional-scale “worst-case-scenario” studies of climate change effects on TCs are indeed consistent with those from the high-resolution global models^23,24^, which also show robust increases of TC intensity and precipitation in a warming climate.

The typhoon damage may vary significantly along the Southeast coast of China, which is impacted by the combined effects from socioeconomic exposures and mitigation measures taken by the coastal provinces. Such regional-scale differences are captured by the ensemble DNN damage models, trained by historical typhoon meteorological data and damage records at a province level. We found that Guangdong Province seems to have lower sensitivity to future typhoon hazard than other coastal provinces. As shown in Fig. 4, for the return period of 50 years, a 20% increase of future typhoon hazard losses would occur compared to the historical damage level. In contrast, all other three typhoon-prone provinces would have an increase of more than 50% in future typhoon-induced losses.

We should expect an uneven risk distribution of typhoon hazard in the future since local populations can adapt to climate change in numerous ways. The province-level DNN models were developed to capture various forms of adaptation to the extent that populations have historically employed them. For example, if local farmers of Guangdong have been adjusting their planting conditions based on recent typhoon activity, the effects of these previous adaptation would be captured by the DNN damage models. Our study applies a “top-down” data-driven macro-level approach that estimates the economic impacts of climate changes without requiring the knowledge of the underlying mechanisms responsible for those losses. Such an approach assumes no future changes of exposure to typhoons and focuses on the direct effects of climate change.

The conventional wind-speed dependent TC damage model has been used to project future TC damage with a high-resolution global climate model^21^ (hereafter G2018). In G2018, the change in mean annual damage due to future storms was projected to increase by about 70% for East Asia, which dominates the global changes. Our estimated 75% increase in the 50-year typhoon damage for all four coastal provinces in China is consistent with G2018. The DNN damage model in our study advances the G2018 study by considering multiple typhoon meteorological variables. The G2018 study does not account for adaptation in the estimates of damage while we capture the adaptation in various provinces with the province-level damage model. Our finding is essentially within the range of those found in existing studies, but our results provide more useful insights for the region of Southeast China, particularly on the changes in potential typhoon-induced damage arising from climate change. On the other hand, our study does not consider future changes in exposure and adaptation. We may overestimate the future typhoon impact as the studies^33,34^ indicate that societies continue to adapt to TCs, so the exposure could be reduced. Despite the limitations, our regional impact study can be used to identify locally appropriate risk reduction (adaptation) measures and provide evidence to facilitate policy and decision making.

A previous study^18^ (hereafter T2019) projected economic impacts of climate change on the global scale considering both climate change mitigation pathways and socioeconomic development pathways^34^, which describe alternate evolutions of the socio-economic system and the eco-environment. T2019 adopted a bottom-up approach by conducting process-based impact simulations for each modeled sector and aggregating the monetized impacts. The economic impact of climate change could be calculated by different impact models for various sectors^18^, e.g., energy, water, transportation, and agriculture. The monetized impacts reported in T2019 were based on only one impact model for each sector; thus the uncertainty associated with variation between the impact models was neglected in T2019. The DNN damage model ensemble developed here is data-driven and are computationally highly efficient. On the other hand, unlike the impact model in T2019 that can be applied more broadly, the data-driven province-level model may be applicable for only the study region in China. Our study does not directly account for TC-induced storm surge, which is expected to increase TC flood hazards under global warming^14,15^, although storm surge is strongly correlated with wind intensity and storm size. Thus, future studies should evaluate the effect of TC-induced storm surge. The effect of different socioeconomic pathways, i.e., sustainable development or fossil-fueled development, may also be investigated. In addition, the DNN damage models could be refined to make independent estimates of economic impact for individual sectors, e.g., agriculture, power, building, etc. The effective and local hazard mitigation measures could then be designed and taken to reduce risks of future typhoon hazards threatening Southeast China.

## Methods

### High-resolution simulations via the PGW approach

The 6-hourly Final (FNL) Operational Global Analysis data at 1° resolution from the National Centers for Environmental Prediction (NCEP) are used for initial and boundary conditions to drive the WRF model for the historical control simulations. We apply a set of parameterization schemes of physical processes through sensitivity experiments for the meso-scale WRF simulation of the 10 SuperTYs to improve the accuracy of simulated TC tracks and intensity. The physical parameterization schemes^35–43^ used in the WRF experiments are also listed in Supplementary Table 6. The microphysics schemes that are used for cloud-resolving simulations include the WRF single-moment 6-class microphysics scheme (WSM6)^40^, New Thompson et al. scheme^41^ (Thompson), and Morrison double-moment scheme^42^ (Morrison). The air-sea flux parameterization^43^, which describes the aerodynamic drag between air and sea, is an important part of the momentum balance in modeling the development of typhoons in the AHW model and is set through the namelist variable of isftcflx (0, 1, 2) corresponding to three different air-sea flux schemes. The ensemble-means of historical typhoon simulations are compared with the observed best track data from the CMA^44,45^, in terms of typhoon tracks and time series of minimum SLP and maximum 10-m wind speed (see Supplementary Figs. 1 and 2).

For the PGW method, atmospheric variables including air temperature, relative humidity, and geopotential height at all tropospheric levels from climate model simulations are used for lateral boundary condition perturbations. The surface variables from climate model simulations, i.e., surface temperature, 2-m air temperature, 2-m specific humidity, SLP, and surface pressure, are used to adjust initial conditions in the PGW experiments. These changed thermodynamic fields are applied to the initial and boundary conditions used in the AHW model to generate “past” or “future” episodes of the 10 SuperTYs. Grid configurations and parameterization schemes of physical processes remain the same as in the historical hindcast simulation for each SuperTY.

### HiFLOR

HiFLOR was developed at the Geophysical Fluid Dynamics Laboratory (GFDL). Seventy-year control HiFLOR simulations^23^ and the future “early” and “late” climate HiFLOR experiments^23,24^ were conducted, and the outputs of these simulations and projections were used in this study. The prescribed climatological SST in the HiFLOR control simulation was the monthly varying climatology from the Met Office Hadley Centre Sea Ice and SST dataset (HadISST1.1)^46^.

We used the HiFLOR “late” experiments to drive the AHW model for the simulations of the 10 SuperTYs in the climate conditions at the end of the twenty-first century under the RCP4.5 scenario. For future typhoon risk estimation, landfalling typhoon events in Southeast China were identified from the HiFLOR “late” RCP4.5 experiments by the TC detection algorithm as described in the literature^23^. In the detection algorithm, SLP and the temperature anomaly averaged between 300 and 500 HPa were the main variables used to locate the local minima of SLP and the warm core of a candidate TC.

Because of the simulation bias of HiFLOR experiments^24^, we applied the bias correction to the frequency of genesis (FOG) and the TC wind speed obtained from the original HiFLOR experiments. Since the FOG bias can be estimated by subtracting observed values from simulated control values in each segmented 2° × 2° grid, the FOG of HiFLOR RCP4.5 experiment could be corrected when assuming that the model biases are inherited by the future projections^31,32^. Supplementary Figure 15 shows the corrected FOG of HiFLOR experiments. The QDM (quantile delta mapping) approach^47^ was applied to adjust the projected maximum 10-m wind speed of HiFLOR. The QDM is commonly used for correcting systematic errors in distributions of a modeled series (HiFLOR data) compared to the control simulation or observation data while preserving model-projected relative changes in quantiles. Supplementary Figure 16 presents the empirical CDFs of maximum 10-m wind speed series from the HiFLOR “late” RCP4.5 experiment, HiFLOR control experiment, the CMA-STI best track dataset, and the corrected HiFLOR “late” projection for Zhejiang, Fujian, Guangdong, and Hainan provinces, respectively.

### Typhoon disaster dataset

Daily precipitation during historical typhoon is taken from the National Meteorological Information Center (http://data.cma.cn/data) of the CMA. The daily area-mean precipitation for a landfalling site is calculated by averaging the daily precipitation from available meteorological stations within the province of the landfalling site. The maximum 2-min sustained wind speed at 10-m height and the minimum SLP of typhoons are derived from the CMA-STI best-track dataset^44,45^. The tropical cyclone size dataset is derived from Satellite Observations^48^. Different data sources have been used to acquire a reliable record of typhoon damages, i.e., direct economic losses, collapsed houses, death tolls, and flooded croplands due to landfalling TCs on coastal provinces of China^49^. The data sources include documents of meteorological disaster for Zhejiang, Fujian, Guangdong, and Hainan provinces^50^, *China Meteorological Yearbook (1986–2017)*^51^, and the *Yearbooks of Meteorological Disaster (2006–2017)*^52^. The overall impact of each observed landfalling typhoon has been quantified in terms of the number of deaths, the number of collapsed houses, the area of flooded croplands, and the direct economic losses (CNY). The direct economic loss is the most important damage measure that is estimated by costs of repair and replacement to restore properties (e.g., houses, farms, works, and roads) to the states before typhoon landfall. We present direct economic losses for the 10 SuperTYs recorded by Department of Civil Affairs of China. The direct economic loss is adjusted by the consumer price index (CPI) of 2013. The CPI data is obtained from the National Bureau of Statistics of China (http://data.stats.gov.cn/search.htm?s=CPI).

### DNN typhoon damage models for predicting direct economic losses

Historical typhoon disaster samples were collected to build the regional-level DNN damage models. The chosen six meteorological variables are used to represent severity of typhoon hazard when the typhoon made landfall, i.e., minimum sea-level pressure, maximum 10-m wind speed, storm size, astronomical tide activity, daily site-maximum precipitation and daily area-mean precipitation from all of the site observations. The astronomical tide index (ASTI) is used to describe astronomical tide activity, which is set as “1” when the typhoon made landfall on the 2nd, 3rd, 17th and 18th day of the lunar calendar. These variables serve as input data to train DNN damage models. Typhoon-induced damage data are represented by direct economic losses. To better train the province-level DNN damage models, the direct economic loss is transformed into economic loss index (ELI) with a range of 0 to 1, which is a normalization of direct economic losses by a given loss threshold, by employing the following conversion function of $U$ as

1 $$U(y) = \left\{ {\begin{array}{ll} {1,} & {10^{12} < y;} \\ {\frac{1}{7}\lg \frac{y}{100000},} & {10^{5} < y \le 10^{12} ;} \\ {0,} & {{\text{y}} \le 10^{5} .} \\ \end{array} } \right.$$

where *y* indicates the direct economic loss adjusted by CPI of 2013 in CNY. In the conversion function of economic loss, we set the upper limit as high as 1000 billion CNY, which might be caused by an unrealistic catastrophic typhoon. Taking Zhejiang Province for example, the typhoon hazard variables associated with 26 historical landfalling typhoon events in a recent 40 years are summarized in Supplementary Table 7, and they serve as input data for training the Zhejiang typhoon damage model. The typhoon damages caused by these TC events in Zhejiang Province are shown in Supplementary Table 8. The ELIs in Supplementary Table 8 are obtained from direct economic losses according to Eq. (1) and served as the output of the typhoon damage model for Zhejiang Province. The maximum ELI of 0.795 for Typhoon Lekima in 2019 corresponds to the direct economic loss of 36.7 billion CNY in Zhejiang Province.

DNNs trained with backpropagation (BP), which is the most representative DNN^53^, has been widely applied in a variety of scientific fields^54–58^. The advantages of DNN models over other regression methods include independent learning and adaptive ability, memory association, and parallel processing of data. The multivariate input–output relationship between landfalling-typhoon characteristics and province-level damages can be readily established in DNN models without explicitly describing the underlining physical and economic theory. Specifically, we develop province-level BP-DNN models for typhoon-prone regions of Southeast China to predict direct economic losses in terms of ELI. Neuron number and hidden layer number are respectively adjusted for each model. For each run, or epoch, the BP-DNN model^59,60^ checks for the mean square error (MSE), or the objective function, and makes efforts to minimize it by altering the biases and weights with a reverse propagation process. The iterations for the optimal weight and bias parameters of the DNN keep going until the MSE decreases to the lowest level or the number of maximum iteration times reaches a predetermined value. To further improve the robustness or the generalization ability of the DNN models, we referred to the committee\ensemble method^61^ to establish ensemble modeling for each province. The way to construct a committee is to average the predictions of a set of individual DNN models. At least 10 DNN models are independently developed to form a model ensemble for predicting typhoon damage for each province.

It is worth noting that possible overfitting could be a problem for the neural network model due to limits of the typhoon damage data. That is why we adopted the committee\ensemble method to alleviate possible overfitting problems. Furthermore, we adjusted the network structure and hyperparameters for each DNN member of the ensemble model, and out-of-sample tests were performed for each DNN damage model. To evaluate the model ensemble, the historical damages of Lekima (Zhejiang), Soudelor (Fujian), Usagi (Guangdong), and Haiyan (Hainan) are then estimated for each province by the corresponding 10-model ensemble and compared to the observed values. The mean of predictions from the model ensemble exhibits a good performance with a maximum error of 4.1% in the case of Soudelor for Fujian Province (see Supplementary Fig. 17a). To demonstrate the robustness of the model, the linear regression of targets (i.e., ELI observations) relative to outputs (i.e., ELI predictions) for 20 testing typhoon samples are presented in Supplementary Fig. 17b.

We further present the ELI results predicted by the DNN model ensembles under three future climate conditions and compare them with the estimated mean ELI from the historical typhoon simulation (see Supplementary Fig. 18). Almost all the 10 typhoon events under a warming climate would produce an increased damage loss when making the first landfall. Particularly, Haiyan, Meranti, and Lekima show significant increases in ELI from the historical events to the future projections under low and high emission scenarios of RCP 4.5 and 8.5. For instance, Typhoon Lekima under HiFLOR RCP4.5 would produce a loss of 64.7 billion CNY in Zhejiang Province, almost 1.8 times of the historical loss of 36.7 billion CNY (CPI-2013 adjusted losses).

### Extreme value analysis for typhoon-induced economic losses

For typhoon risk analysis, we need to consider adequate samples of typhoon events and their damage data. The HiFLOR “late” experiments are able to project future intense storms for the WNP basin considering the climate during 2080–2100 under the RCP4.5 scenario. The outputs of the HiFLOR experiments (bias-corrected) serve as inputs for the DNN typhoon damage models to estimate the typhoon-induced loss in the future climate. The maximum 2-min sustained wind speed at 10-m height, the minimum SLP, daily site-maximum, and daily area-mean precipitation of serve as predicting variables in the DNN typhoon damage models to predict future typhoon damage of each future typhoon sample from the HiFLOR RCP4.5 experiment.

We apply the same extreme value analysis to both the historical typhoon damage data and the predicted damage data of future typhoons. Since the future typhoon damage is predicted using the corrected HiFLOR “late” projection data, differences between historical typhoon damage and the predicted future damage as presented in Fig. 4 could be attributed to the effects of anthropogenic warming. Return period curves of economic loss are obtained for Zhejiang, Fujian, Guangdong, and Hainan provinces, respectively, as shown in Fig. 4. Assuming that the annual maximum economic loss distribution conforms to the generalized extreme value (GEV) distribution, the return period T of TC-induced economic losses is defined as^62,63^:

2 $${\text{T}} = \frac{1}{{1 - F(y_{{\text{m}}} )}}$$

where $F(y_{{\text{m}}} )$ is the cumulative probability distribution (CDF) of annual maximum economic loss. We model the tail of the future typhoon damage data using the Peaks-Over-Threshold method with a generalized Pareto distribution^64^. We determine the economic loss threshold value for each province to separate the tail from the rest of the distribution by trial and error to achieve the overall minimum error of tail fitting. The CDF of the generalized Pareto distribution with the shape parameter *k*, the scale parameter *σ*, and the threshold parameter *μ*, is given as^64^:

3 $$\left\{ {G(y_{{\text{m}}} ) = 1 - \left( {1 + k\frac{{y_{{\text{m}}} - \mu }}{\sigma }} \right)^{{ - {1 \mathord{\left/ {\vphantom {1 k}} \right. \kern-\nulldelimiterspace} k}}} ,\;\;\,y_{{\text{m}}} \ge \mu , \, \left( {1 + k\frac{{y_{{\text{m}}} - \mu }}{\sigma }} \right) > 0} \right\}$$

## Supplementary Information

Supplementary Information.

## Data Availability

The datasets used and/or analyzed during the current study are available from the corresponding author on reasonable request.
