# Supplementary material for: Increasing typhoon impact and economic losses due to anthropogenic warming in Southeast China
Source: Sci Rep. 2022 Sep 8;12:14048. doi: 10.1038/s41598-022-17323-8 (PMC9458652; doi:10.1038/s41598-022-17323-8)
Supplement: Supplementary file 1 — Supplementary Information. [file 41598_2022_17323_MOESM1_ESM.docx]

Supplementary Information for

**Increasing typhoon impact and economic losses due to anthropogenic warming in Southeast China**

Mingfeng Huang^1,2^, Qing Wang^1^, Maofeng Liu^3^, Ning Lin^4*^, Yifan Wang^1^, Renzhi Jing^4^, Jianping Sun^1^, Hiroyuki Murakami^5^, Wenjuan Lou^1^

*^1^Institute of Structural Engineering, College of Civil Engineering and Architecture, Zhejiang University, Hangzhou* 310058, *China*

*^2^Shanghai Institute for Advanced Study, Zhejiang University, Shanghai, 201203, China*

*^3^Rosenstiel School of Marine and Atmospheric Science, University of Miami, Miami, USA*

^4^Department *of Civil and Environmental Engineering, Princeton University, Princeton, New Jersey, USA. *e-mail:* nlin@princeton.edu

*^5^NOAA/Geophysical Fluid Dynamics Laboratory, and Atmospheric and Oceanic Sciences Program, Princeton University, Princeton, New Jersey, USA*

**Supplementary Tables.**

**Supplementary Table 1:** Ten landfalling major typhoons in 2013-2019.

| TC name | TC No. | Historical simulation period | Landing location | Affected provinces |
| --- | --- | --- | --- | --- |
| Usagi | 1319* | 17-23 Sept., 2013 | Guangdong | Guangdong,  Fujian |
| Haiyan | 1330 | 5 - 11 Nov, 2013 | Philippines; Vietnam; Guangxi, China | Guangdong, Guangxi, Hainan |
| Rammasun | 1409 | 10-21 July, 2014 | Hainan, China | Haian, Guangdong, Guangxi |
| Chan-hom | 1509 | 8 - 12 Jul, 2015 | North Korea | Zhejiang, Jiangsu |
| Soudelor | 1513 | 30 July- 1 Aug., 2015 | Fujian, China | Fujian, Zhejiang, Jiangsu |
| Mujigae | 1522 | 3 - 5 Oct, 2015 | Guangdong, China | Guangdong, Guangxi |
| Meranti | 1614 | 11 - 15 Sep, 2016 | Fujian, China | Fujian, Zhejiang |
| Maria | 1808 | 8 - 11 Jul, 2018 | Fujian, China | Fujian, Zhejiang |
| Mangkut | 1822 | 13 - 17 Sep, 2018 | Guangdong, China | Guangdong, Guangxi |
| Lekima | 1909 | 7 - 13 Aug, 2019 | Zhejiang, China | Zhejiang, Jiangsu |

*From 1996, a four-digit serial number was added to each typhoon. The first two digits represent the year, the last two the sequence of the typhoon in the year. For example, the name "Typhoon 1319 Usagi" means that the typhoon Usagi occurred in 2013 in WNP and it was the nineteenth typhoon of that year.

**Supplementary Table 2**: Statistical disaster data of recent landfalling SuperTYs in China

| Typhoon | Province | Collapsed Houses (room) | Flooded  Croplands (1,000 hectares) | Death tolls (Person) | Direct economic losses (million CNY) | Adjusted direct economic losses (million CNY)^*^ |
| --- | --- | --- | --- | --- | --- | --- |
| 1319  Usagi | Guangdong | 11000 | 255 | 30 | 23550 | 23550 |
|  | Hunan | 1000 | 101 | 4 | 670 | 670 |
|  | Fujian | 1000 | 67 | / | 2070 | 2070 |
|  | Guangxi | / | 4 | / | 90 | 90 |
|  | Jiangxi | / | / | / | 20 | 20 |
|  | **Total** | **13000** | **337** | **34** | **26400** | **26400** |
| 1330 Haiyan | Hainan | 1000 | 134 | 13 | 3050 | 3050 |
|  | Guangxi | 4000 | 340 | 7 | 1440 | 1440 |
|  | Guangdong | / | 81 | / | 90 | 90 |
|  | **Total** | **5000** | **555** | **20** | **4580** | **4580** |
| 1409  Rammasun | Hainan | 25000 | 163 | 26 | 11910 | 11676 |
|  | Guangdong | 7311 | 226 | / | 15340 | 15040 |
|  | Guangxi | 10000 | 775 | 10 | 13990 | 13716 |
|  | Yunnan | 4000 | 100 | 37 | 3410 | 3343 |
|  | **Total** | **46311** | **1264** | **73** | **44650** | **43775** |
| 1509 Chan-hom | Zhejiang | 3500 | 329 | / | 8860 | 8567 |
|  | Shanghai | / | 8 | / | 230 | 222 |
|  | Jiangsu | 100+ | 54 | / | 220 | 213 |
|  | Anhui | / | 5 | / | 30 | 29 |
|  | Shandong | / | 6 | / | 10 | 10 |
|  | **Total** | **1100+** | **293** | **/** | **9590** | **9272** |
| 1513 Soudelor | Zhejiang | 2000 | 92 | 15 | 11080 | 10713 |
|  | Fujian | 5000 | 104 | 8 | 7880 | 7619 |
|  | Anhui | 3000 | 94 | 4 | 3150 | 3046 |
|  | Jiangxi | 1000 | 40 | 1 | 620 | 599 |
|  | Jiangsu | 1000 | 205 | / | 1520 | 1470 |
|  | **Total** | **12000** | **535** | **28** | **24250** | **23446** |
| 1522  Mujigae | Guangdong | 7000 | 521 | 18 | 27070 | 26172 |
|  | Guangxi | 3000 | 161 | 1 | 1770 | 1711 |
|  | Hainan | / | 34 | 1 | 1170 | 1131 |
|  | **Total** | **10000** | **716** | **20** | **30010** | **29015** |
| 1614 Meranti | Fujian | 10000 | 73 | 31 | 26190 | 24825 |
|  | Zhejiang | 2000 | 46 | 7 | 5430 | 5147 |
|  | Jiangxi | / | / | / | 2 | 2 |
|  | Shanghai | / | 3 | / | 20 | 19 |
|  | Jiangsu | / | / | / | 1 | 1 |
|  | **Total** | **12000** | **122** | **38** | **31640** | **29991** |
| 1808 Maria | Zhejiang | / | 17 | / | 710 | 650 |
|  | Fujian | 300 | 28 | / | 2720 | 2490 |
|  | Jiangxi | / | 5 | 1 | 35 | 32 |
|  | Hunan | / | 2 | / | 14 | 13 |
|  | **Total** | **300** | **50** | **1** | **3279** | **3005** |
| 1822 Mangkhut | Guangdong | 100 | 104 | 5 | 3210 | 2939 |
|  | Guangxi | 700 | 51 | / | 180 | 165 |
|  | Hainan | / | / | / | / | / |
|  | Hunan | 30 | / | / | 1 | 1 |
|  | Guizhou | 10 | / | / | / | / |
|  | **Total** | **900** | **154** | **5** | **3401** | **3113** |
| 1909 Lekima | Liaoning | 800 | 25 | / | 79 | 71 |
|  | Jilin | / | 14 | / | 28 | 25 |
|  | Hebei | 500 | 17 | / | 84 | 75 |
|  | Fujian | 100 | / | / | 2 | 2 |
|  | Shanghai | / | 3 | / | 45 | 41 |
|  | Jiangsu | 400 | 155 | 1 | 460 | 415 |
|  | Zhejiang | 6300 | 258 | 45 | 40720 | 36712 |
|  | Anhui | 1900 | 24 | 5 | 3290 | 2966 |
|  | Shandong | 6700 | 643 | 5 | 9010 | 8123 |
|  | **Total** | **16000** | **1140** | **57** | **53718** | **48430** |

*The direct economic losses data was adjusted by consumer price index (CPI) of 2013.

**Supplementary Table 3:** List of 10 CMIP5 models

| Model | Affiliation, country | Resolution(°) | Pressure level |
| --- | --- | --- | --- |
| ACCESS 1.3 | CSIRO-BOM, Australia | 1.88×1.24 | 17 |
| CanESM2 | CCCMA, Canada | 2.81×2.81 | 22 |
| CNRM-CM5 | CNRM-CERFACS, France | 1.41×1.41 | 17 |
| GFDL-CM3 | NOAA GFDL, USA | 2.5×2 | 17 |
| GFDL-ESM2M | NOAA GFDL, USA | 2.5×2 | 17 |
| HadGEM2-AO | NIMR/KMA, South Korea/England | 1.88×1.24 | 17 |
| MIROC5 | MIROC, Japan | 1.41×1.41 | 17 |
| MRI-CGCM3 | MRI, Japan | 1.13×1.13 | 23 |
| CCSM4 | NCAR/UCAR, USA | 1.25×0.94 | 26 |
| FGOALS-s2 | LASG-IAP, China | 2.81×1.67 | 17 |

**Supplementary Table 4**: Minimum sea-level pressure at landfall (hPa)

| Typhoon | Observed | Historical  hindcasts  (mean±$\sigma)$ | RCP4.5 minus Historical | RCP8.5 minus  Historical | RCP4.5 (HiFLOR) minus  Historical |
| --- | --- | --- | --- | --- | --- |
| Usagi | 930.0 | 933.6 ±1.1 | -1.7 | -6.1 | -5.2 |
| Haiyan | 960.0 | 955.2 ±2.7 | -6.7 | -9.8 | -7.7 |
| Rammasun | 910.0 | 917.6 ±4.6 | -0.8 | -15.7 | -3.7 |
| Chan-hom | 955.0 | 940.0 ±2.9 | -3.8 | -9.3 | -4.9 |
| Soudelor | 965.0 | 944.9 ±3.5 | -5.0 | -11.2 | -5.7 |
| Mujigae | 935.0 | 948.3 ±4.3 | -5.9 | -17.4 | -6.0 |
| Meranti | 940.0 | 947.5 ±4.4 | -8.5 | -9.7 | -8.9 |
| Maria | 955.0 | 945.6 ±4.4 | -3.8 | -11.9 | -7.8 |
| Mangkut | 950.0 | 932.5 ±4.1 | -2.9 | -10.4 | -6.5 |
| Lekima | 925.0 | 915.8 ±2.9 | -6.6 | -8.7 | -16.2 |

**Supplementary Table 5:** Daily area-mean precipitation at landfall

| Typhoon | Observed | Historical  hindcasts  (mean±$\sigma)$ | RCP4.5 minus Historical | RCP8.5 minus  Historical | RCP4.5 (HiFLOR) minus  Historical |
| --- | --- | --- | --- | --- | --- |
| Usagi | 18.0 | 80.4 ±4.2 | 29.7 | 22.6 | -3.9 |
| Haiyan | 100.0 | 53.9 ±32.4 | 22.5 | 26.6 | 64.5 |
| Rammasun | 45.7 | 59.1 ±10.5 | -8.5 | 1.7 | -8.5 |
| Chan-hom | 65.4 | 33.1 ±15.2 | 3.7 | 10.1 | 35.0 |
| Soudelor | 47.3 | 65.2 ±5.2 | 11.1 | 26.6 | 8.0 |
| Mujigae | 54.0 | 35.1 ±4.8 | 16.3 | 20.1 | 16.7 |
| Meranti | 62.6 | 44.0 ±11.7 | 6.6 | 19.6 | 13.6 |
| Maria | 34.5 | 33.7±8.5 | -3.2 | 2.2 | 1.4 |
| Mangkut | 55.8 | 43.0 ±4.8 | 6.3 | 7.5 | 2.5 |
| Lekima | 101.2 | 124.0 ±16.1 | 7.3 | 14.6 | 7.1 |

**Supplementary Table 6**: Summary of grid configuration and physical parameterization in AHW experiments for the 10 SuperTYs.

| TC | Grid length (km) | Grid number | Time step | Microphysics | isftcflx |
| --- | --- | --- | --- | --- | --- |
| Usagi | 12, 4 | 199×142, 136×115 | 60, 20 | Morrison | 2 |
| Haiyan | 36,12,4 | 191×108, 145×106,  199×181 | 120, 40, 40/3 | Morrison | 0 |
| Rammasun | 12,4 | 253×194, 154×139 | 60, 20 | Thompson | 1 |
| Chan-hom | 36,12,4 | 162×131, 226×232,  211×217 | 180, 60, 20 | Morrison | 1 |
| Soudelor | 12,4 | 258×185, 160×154 | 60, 20 | WSM6 | 0 |
| Mujigae | 36,12,4 | 138×104, 190×145,  148×124 | 180, 60, 20 | Morrison | 1 |
| Meranti | 36,12,4 | 179×136, 271×196,  196×178 | 120, 40, 40/3 | Thompson | 1 |
| Maria | 36,12,4 | 161×112, 151×127,  160×145 | 180, 60, 20 | Morrison | 0 |
| Mangkut | 36,12,4 | 199×110, 121×118,  163×136 | 120, 40, 40/3 | Thompson | 0 |
| Lekima | 12,4 | 211×229, 139×109 | 60, 20 | Thompson | 1 |

*The Dudhia scheme^32^ and RRTM scheme^33^ were adopted for short-wave and long wave radiation, respectively. The Kain-Fritsch (new Eta) cumulus scheme^34^ was activated for relatively larger domains (36, 12 km) and the YSU planetary boundary layer^35^ (PBL) schemes were adopted. The surface layer scheme of Revised MM5 Monin–Obukhov similarity^36^ was used together with the surface boundary conditions of the Unified Noah land surface model.

**Supplementary Table 7**: Typhoon hazard variables for DNN model of Zhejiang Province.

| Typhoon number | ASTI | Maximum 10-m wind speed at landfall (m/s) | Minimum sea-level pressure at landfall (hPa) | storm size at landfall (km) | Daily site-maximum precipitation at landfall (mm) | Daily area-mean precipitation at landfall (mm) |
| --- | --- | --- | --- | --- | --- | --- |
| 8108 | 0 | 20 | 996 | 189.6 | 101.5 | 34.6 |
| 8403 | 0 | 25 | 998 | 222.6 | 56.2 | 10.4 |
| 8506 | 0 | 40 | 968 | 215.6 | 195.5 | 29.2 |
| 8707 | 1 | 35 | 970 | 193.2 | 131 | 22.9 |
| 8807 | 0 | 35 | 975 | 139.0 | 139.3 | 39.7 |
| 8909 | 1 | 30 | 978 | 183.2 | 241.5 | 37.2 |
| 9417 | 1 | 45 | 958 | 200.4 | 216.3 | 46.8 |
| 9507 | 0 | 30 | 990 | 162.8 | 147 | 35.1 |
| 9711 | 1 | 40 | 962 | 220.3 | 199.9 | 59.1 |
| 0311 | 0 | 23 | 991 | 152.0 | 66.9 | 22.9 |
| 0414 | 0 | 45 | 950 | 228.6 | 171.2 | 40.5 |
| 0421 | 0 | 18 | 997 | 0 | 121.3 | 43.8 |
| 0509 | 0 | 45 | 957 | 199.5 | 155.5 | 65.9 |
| 0515 | 0 | 50 | 945 | 201.3 | 260.5 | 60.9 |
| 0608 | 1 | 60 | 940 | 211.1 | 374.0 | 30.5 |
| 0713 | 0 | 45 | 955 | 205.3 | 141.3 | 68.9 |
| 0716 | 0 | 28 | 985 | 142.1 | 184.6 | 69.1 |
| 0807 | 0 | 20 | 995 | 111.12 | 83.3 | 17.3 |
| 0903 | 0 | 18 | 995 | 0 | 41.6 | 4.5 |
| 0908 | 0 | 25 | 986 | 168.3 | 146.8 | 46.6 |
| 1010 | 1 | 20 | 998 | 83.3 | 38.2 | 11.1 |
| 1211 | 0 | 42 | 973 | 204.1 | 198.2 | 82.5 |
| 1416 | 0 | 25 | 987 | 111.1 | 137.3 | 33.1 |
| 1509 | 0 | 42 | 955 | 176.8 | 267.7 | 47.3 |
| 1812 | 0 | 23 | 985 | 111.0 | 134.9 | 32.0 |
| 1909 | 0 | 52 | 925 | 250.0 | 291.0 | 101.2 |

**Supplementary Table 8:** Historical typhoon loss data of recent 40 years in Zhejiang Province.

| Typhoon number | Typhoon  name | Collapsed Houses (room) | Flooded  Croplands (1,000 hectares) | Death tolls (Person) | Adjusted direct economic losses (million CNY)^*^ | ELI |
| --- | --- | --- | --- | --- | --- | --- |
| 8108 | (nameless) | 500 | 22.9 | 2 | 29 | 0.352 |
| 8403 | Alex | 100 | 2.1 | 4 | 6 | 0.258 |
| 8506 | Jeff | 23500 | 220.2 | 232 | 1425 | 0.593 |
| 8707 | Alex | 15700 | 189.3 | 116 | 2239 | 0.621 |
| 8807 | Bill | 66900 | 218.0 | 162 | 3778 | 0.654 |
| 8909 | Hope | 53800 | 184.0 | 132 | 3627 | 0.651 |
| 9417 | Fred | 208300 | 502.0 | 1126 | 31161 | 0.785 |
| 9507 | Janis | 300 | 60.7 | 0 | 124 | 0.442 |
| 9711 | Winnie | 177000 | 747.3 | 239 | 25036 | 0.771 |
| 0311 | Vamco | 1000 | 6.8 | 0 | 15 | 0.311 |
| 0414 | Rananim | 64300 | 391.9 | 179 | 23656 | 0.768 |
| 0421 | Haima | 0 | 7.8 | 0 | 391 | 0.513 |
| 0509 | Matsa | 19000 | 339.0 | 5 | 11422 | 0.723 |
| 0515 | Khanun | 23000 | 317.0 | 23 | 15562 | 0.742 |
| 0608 | Saomai | 53200 | 103.2 | 204 | 16085 | 0.744 |
| 0713 | Wipha | 4900 | 253.0 | 8 | 6772 | 0.690 |
| 0716 | Krosa | 5100 | 422.0 | 0 | 10737 | 0.719 |
| 0807 | Kalmaegi | 0 | 4.3 | 0 | 73 | 0.409 |
| 0903 | Linfa | 0 | 1.1 | 0 | 49 | 0.385 |
| 0908 | Morakot | 7700 | 350.4 | 5 | 10135 | 0.715 |
| 1010 | Meranti | 0 | 12.6 | 0 | 144 | 0.451 |
| 1211 | Haikui | 5000 | 366.0 | 0 | 28001 | 0.778 |
| 1416 | Fung-wong | 0 | 57.0 | 0 | 931 | 0.567 |
| 1509 | Chan-hom | 3500 | 329.3 | 0 | 8567 | 0.705 |
| 1812 | Jongdari | 0 | 12.7 | 0 | 339 | 0.504 |
| 1909 | Lekima | 6300 | 258.4 | 45 | 36712 | 0.795 |

*The direct economic losses data was normalized by consumer price index (CPI) of 2013.

**Supplementary Figures**

**(b)**

**(a)**

**(d)**

**(c)**

**(f)**

**(e)**

**(h)**

**(g)**

**(i)**

**Supplementary Figure 1:** Tracks for Typhoon Usagi (**a**), Haiyan (**b**), Rammasun (**c**), Chan-hom (**d**), Soudelor **(e**), Mujigae (**f**), Meranti (**g**), Maria (**h**) and Mangkhut (**i**). Observations (black), ensemble mean of the pre-industrial (blue), historical (gray), RCP4.5 of HiFLOR (red), RCP4.5 (green) and 8.5 (purple) of CMIP5 mean simulations at 4-km resolution. Figures were produced by MATLAB R2021a (http://www.mathworks.com/).

**(b)**

**(a)**

 ****

**(d)**

**(c)**

**(f)**

**(e)**

**(h)**

**(g)**

 ****

**(j)**

**(i)**

**(k)**

**(l)**

**(n)**

**(m)**

**(p)**

**(o)**

**(r)**

**(q)**

**Supplementary Figure 2:** Time series of minimum SLP and maximum 10-m wind speed for Typhoon Usagi (**a, b**), Haiyan (**c, d**), Rammasun (**e, f**), Chan-hom (**g, h**), Soudelor **(i, j**), Mujigae (**k, l**), Meranti (**m, n**), Maria (**o, p**) and Mangkhut (**q, r**). Observations (black), ensemble mean of the pre-industrial (blue), historical (gray), RCP4.5 of HiFLOR (red), RCP4.5 (green) and 8.5 (purple) of CMIP5 mean simulations at 4-km resolution. Observed landing time was marked with a vertical gray dashed line in (**b, d, f, h, j, l, n, p, r**).


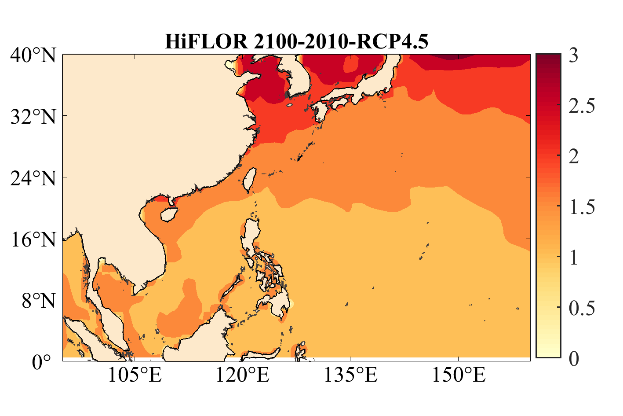


**Supplementary Figure 3:** Increments of SST between future and historical climate from HiFLOR RCP 4.5. Maps were generated by MATLAB R2021a (http://www.mathworks.com/).


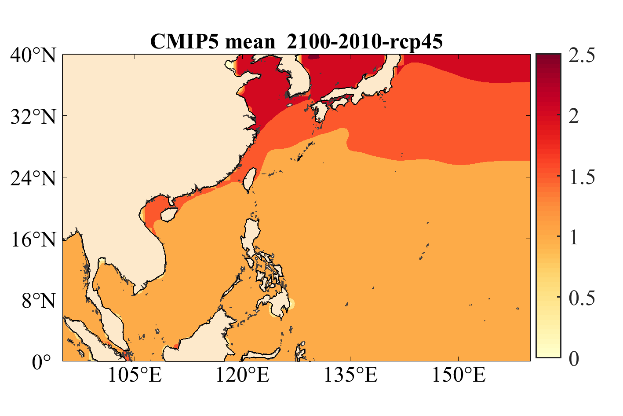

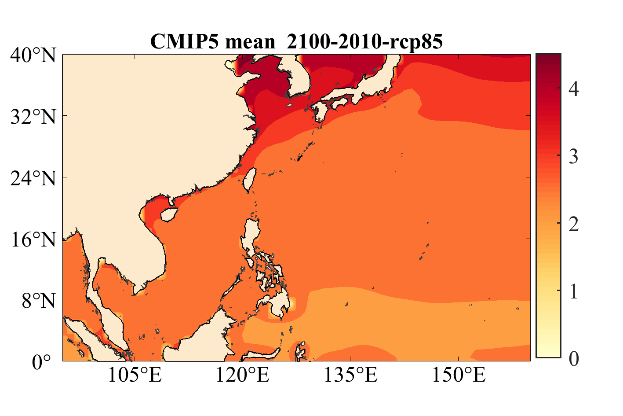


**(b)**

**(a)**


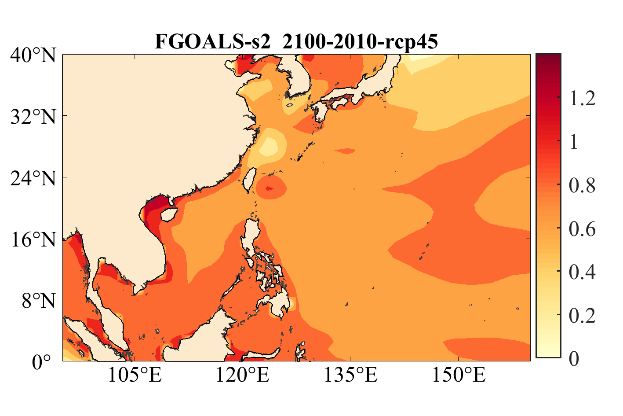

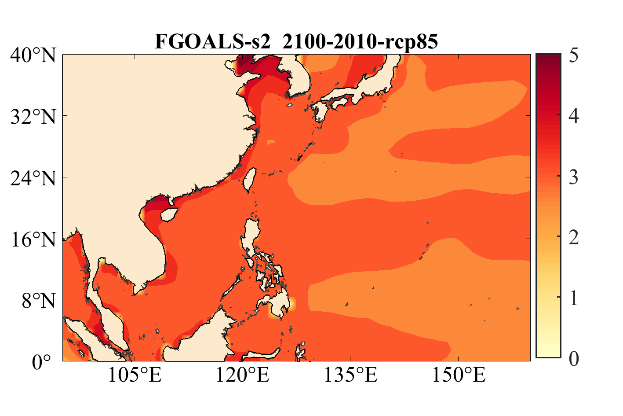


**(d)**

**(c)**

**
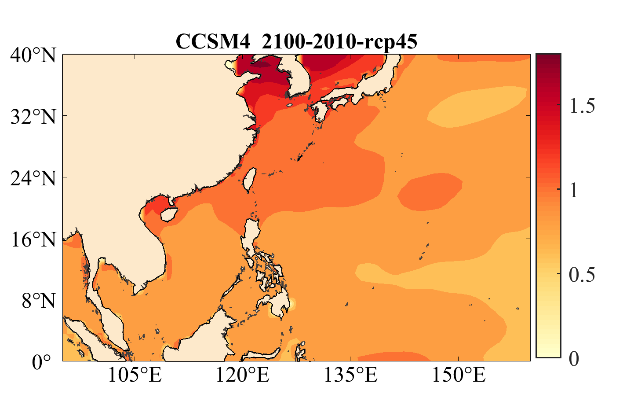

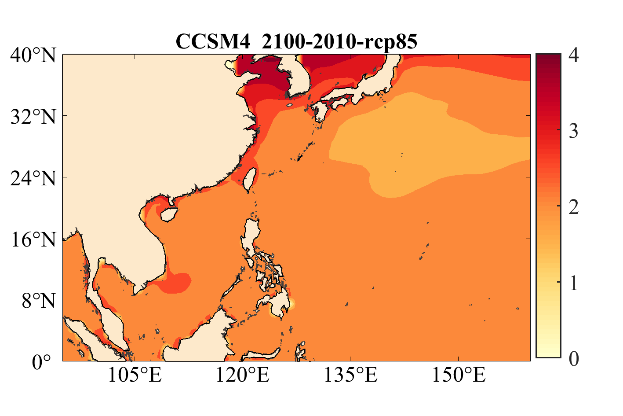
**

**(f)**

**(e)**

**Supplementary Figure 4:** Increments of SST between historical and future climate from the CMIP5 ten-model mean (**a**, **b**), FGOALS-s2 (**c**, **d**) and CCSM4 model (**e**, **f**) under (**a**, **c**, **e**) RCP 4.5 and (**b**, **d**, **f**) RCP 8.5 emission scenarios. Maps were generated by MATLAB R2021a (http://www.mathworks.com/).


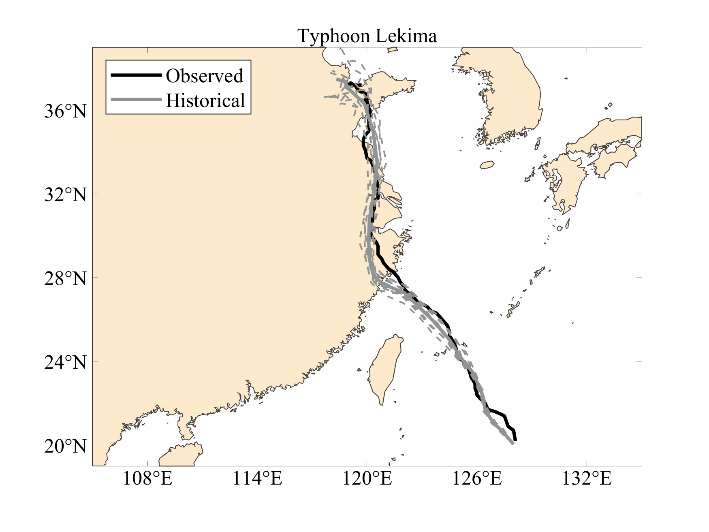


**(a)**


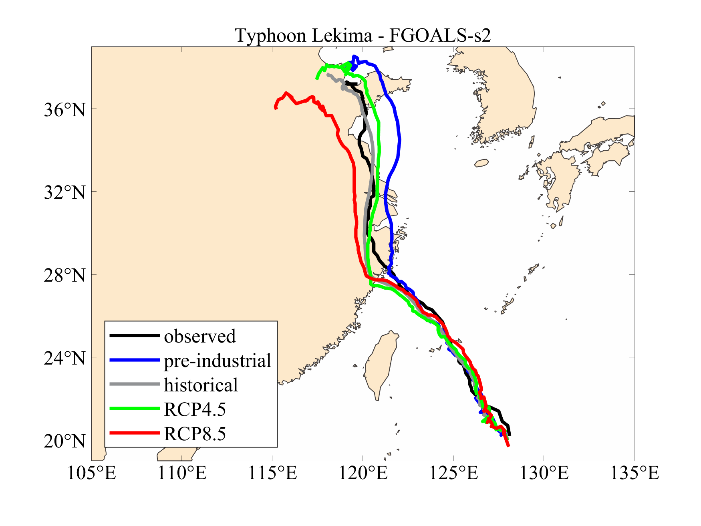

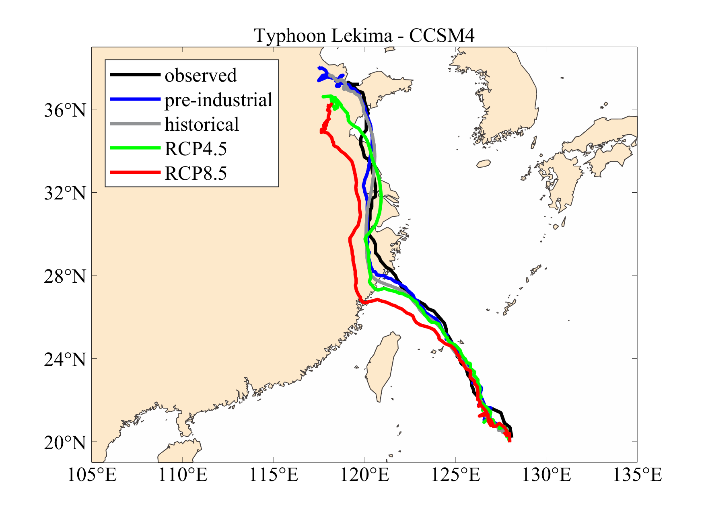


**(c)**

**(b)**

**Supplementary Figure 5:** Typhoon Lekima tracks. Observed tracks (black) with simulated TC tracks from ten ensemble members (grey dashed line) (**a**) and the ensemble mean track (grey line) of the historical simulation (**a**-**c**). Pre-industrial simulation (1860-1880) and future typhoon simulation (2080-2100) under RCP 4.5 and RCP 8.5 emission scenarios corresponding to FGOALS-s2 (**b**) and CCSM4 model (**c**). Maps were generated by MATLAB R2021a (http://www.mathworks.com/).


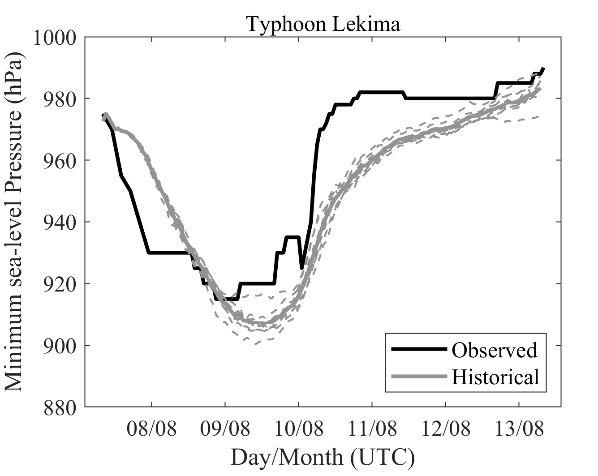


**(b)**

**(a)**


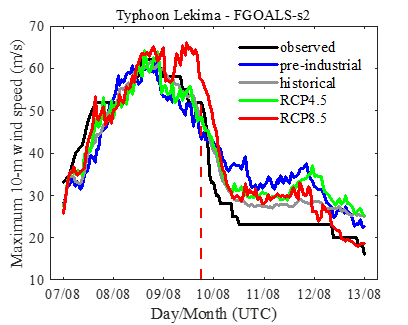

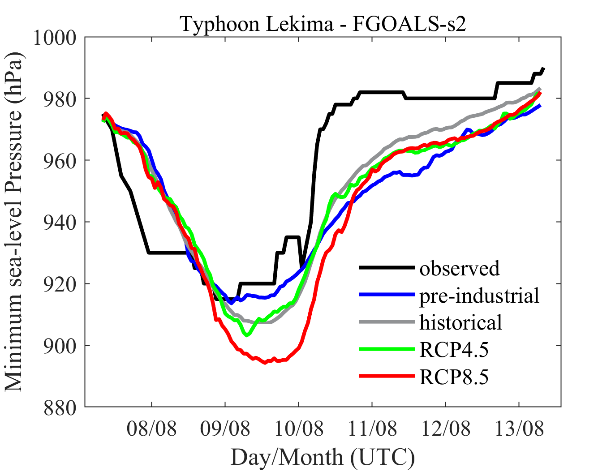


**(d)**

**(c)**


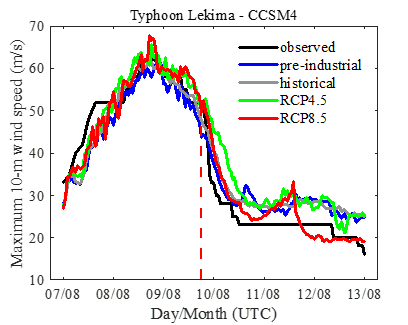

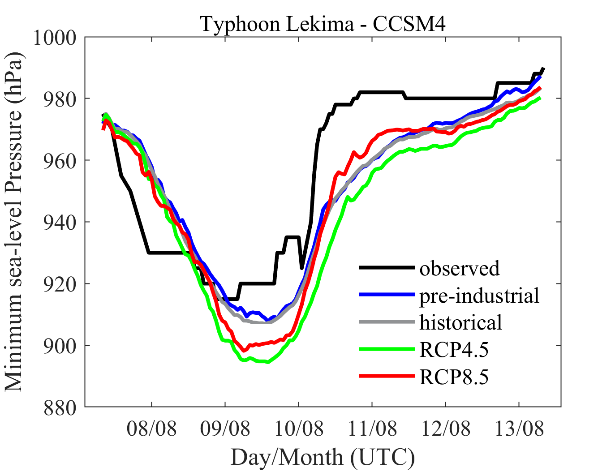


**(f)**

**(e)**

**Supplementary Figure 6:** Time series of (**a**, **c**, **e**) maximum 10-m wind speed and (**b**, **d**, **f**) minimum sea-level central pressure of Typhoon Lekima. Historical simulation (2000-2020), pre-industrial simulation (1860-1880) and future typhoon simulation (2080-2100) under RCP 4.5 and RCP8.5 emission scenarios corresponding to FGOALS-s2 (**c**, **d**) and CCSM4 model (**e**, **f**). Observed landing time was marked with a vertical red dashed line in a, c, e.


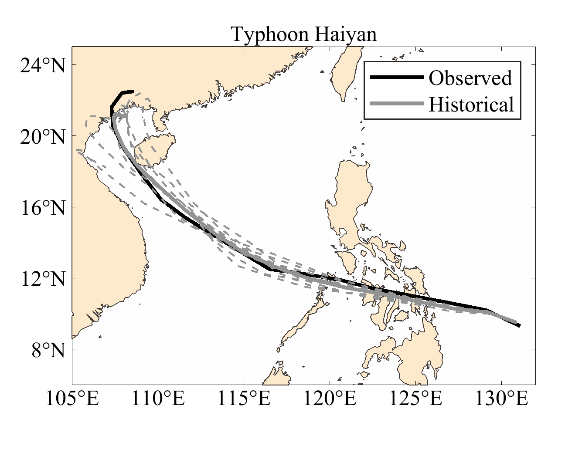


**(a)**


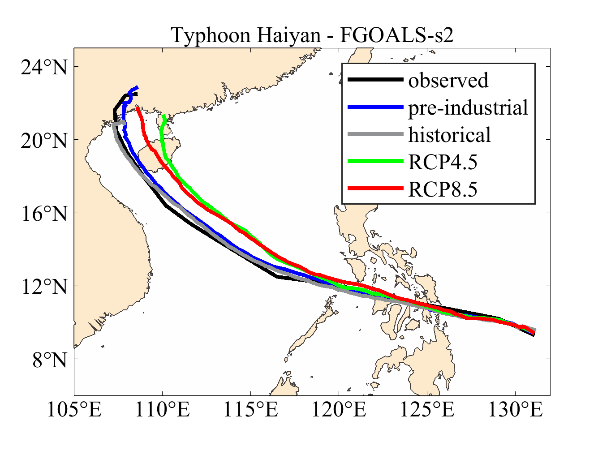

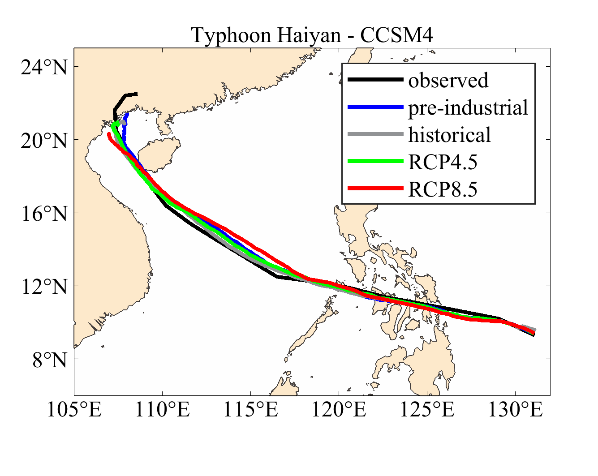


**(c)**

**(b)**

**Supplementary Figure 7:** Typhoon Haiyan tracks. Observed tracks (black) with simulated TC tracks from ten ensemble members (grey dashed line) (**a**) and the ensemble mean track (grey line) of the historical simulation (**a**-**c**). Pre-industrial simulation (1860-1880) and future typhoon simulation (2080-2100) under RCP 4.5 and RCP 8.5 emission scenarios corresponding to FGOALS-s2 (**b**) and CCSM4 model (**c**). Maps were generated by MATLAB R2021a (http://www.mathworks.com/).


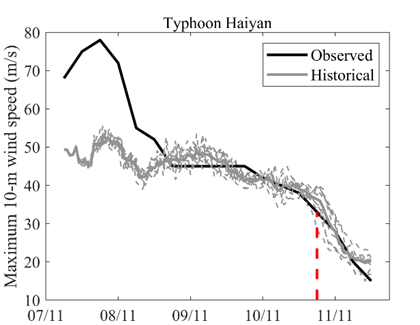

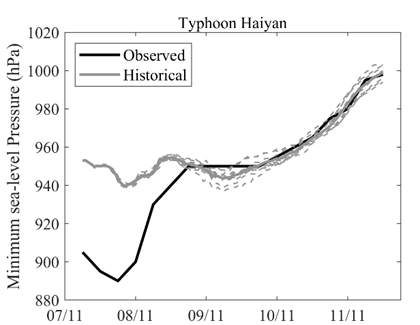


**(b)**

**(a)**


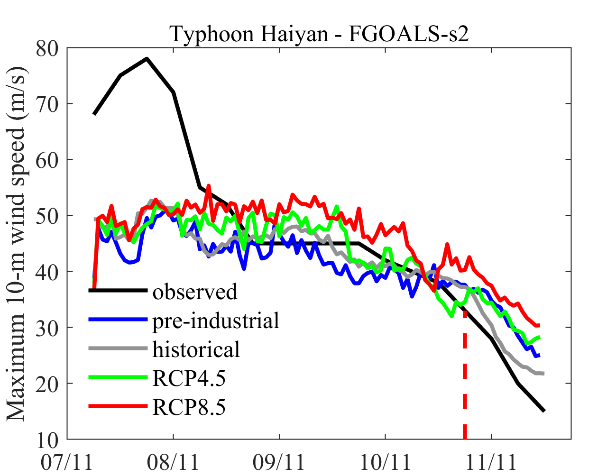

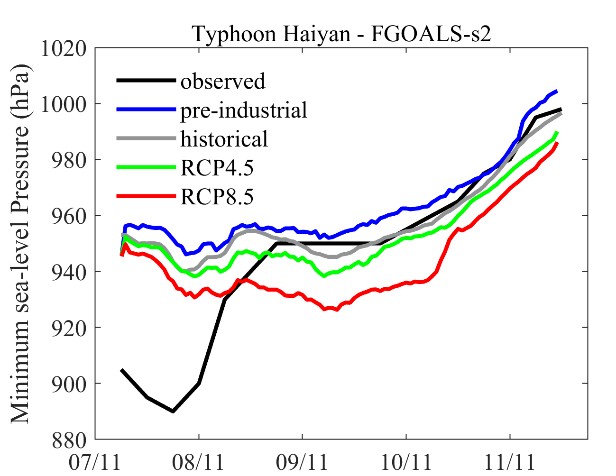


**(d)**

**(c)**


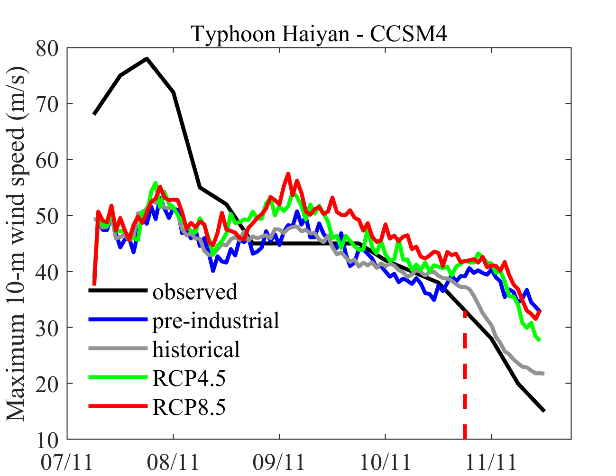

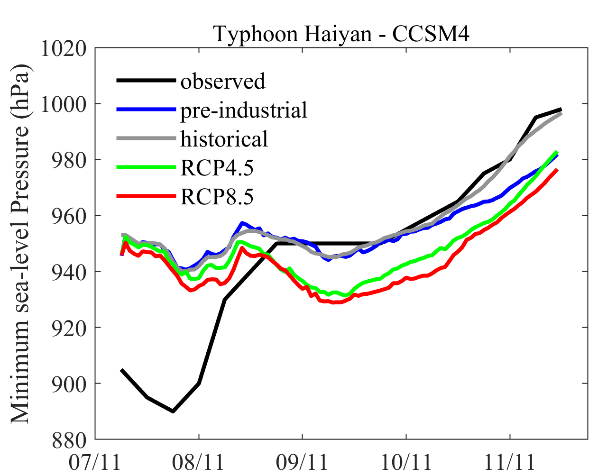


**(f)**

**(e)**

**Supplementary Figure 8:** Time series of (**a**, **c**, **e**) maximum 10-m wind speed and (**b**, **d**, **f**) minimum sea-level central pressure of Typhoon Haiyan. Historical simulation (2000-2020), pre-industrial simulation (1860-1880) and future typhoon simulation (2080-2100) under RCP 4.5 and RCP8.5 emission scenarios corresponding to FGOALS-s2 (**c**, **d**) and CCSM4 model (**e**, **f**). Observed landing time was marked with a vertical red dashed line in **a**, **c**, **e**.


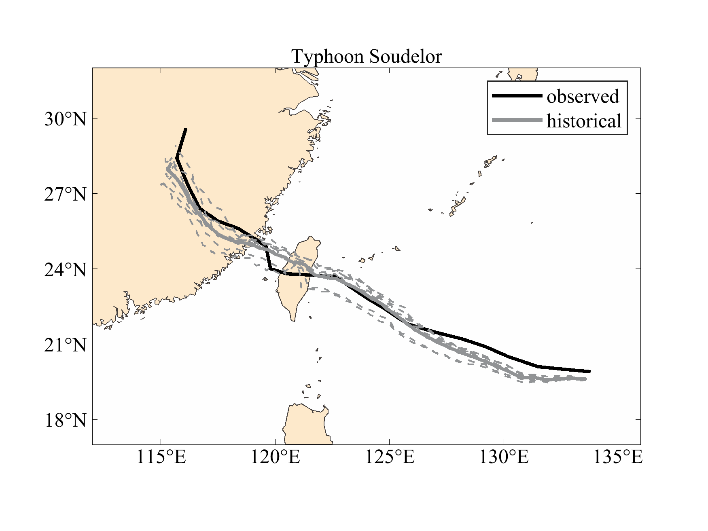


**(a)**


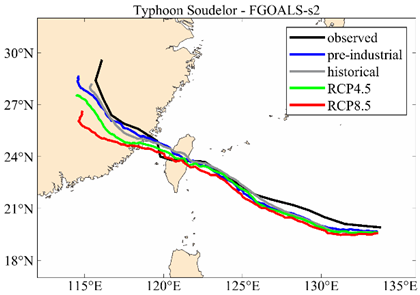

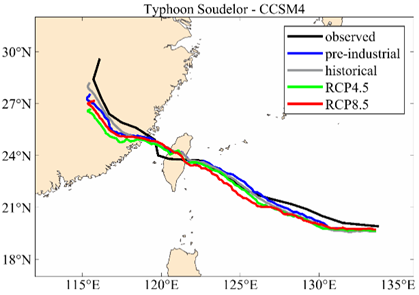


**(c)**

**(b)**

**Supplementary Figure 9:** Typhoon Souldelor tracks. Observed tracks (black) with simulated TC tracks from ten ensemble members (grey dashed line) (**a**) and the ensemble mean track (grey line) of the historical simulation (**a**-**c**). Pre-industrial simulation (1860-1880) and future typhoon simulation (2080-2100) under RCP 4.5 and RCP 8.5 emission scenarios corresponding to FGOALS-s2 (**b**) and CCSM4 model (**c**). Maps were generated by MATLAB R2021a (http://www.mathworks.com/).


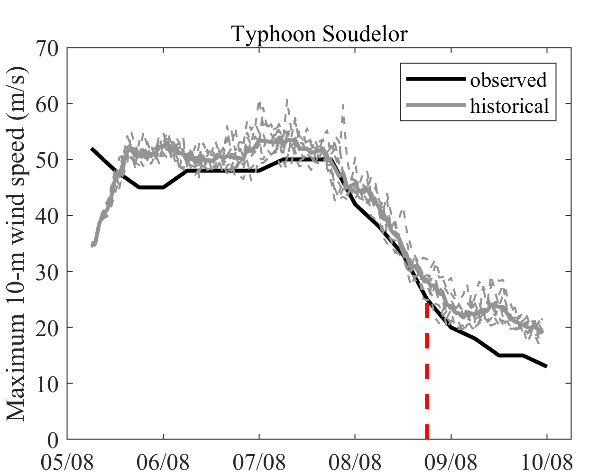

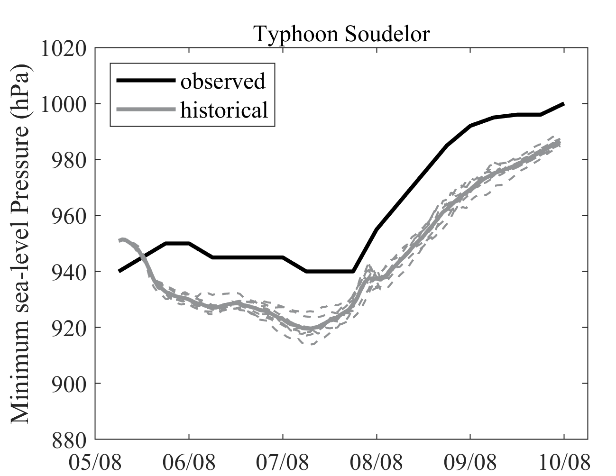


**(b)**

**(a)**


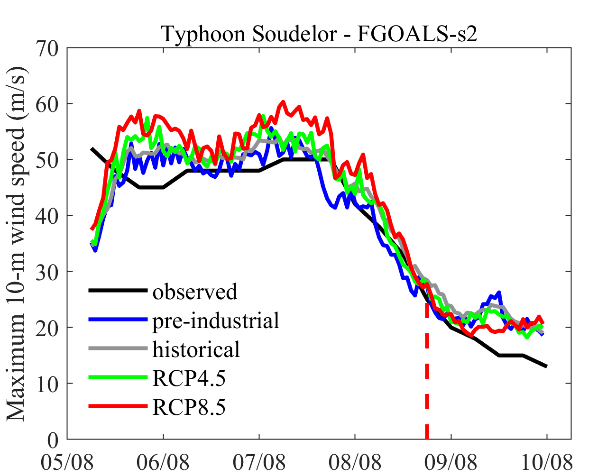

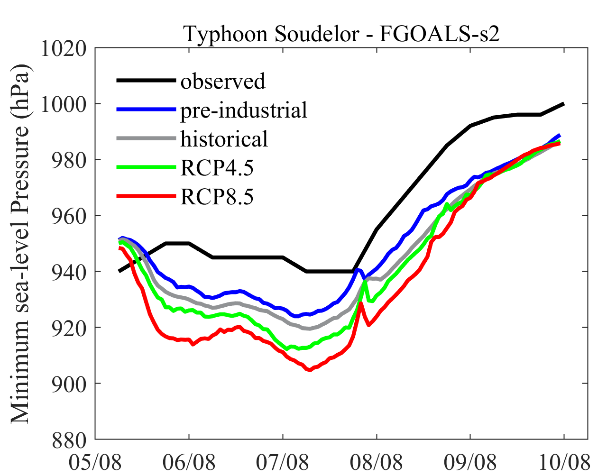


**(d)**

**(c)**


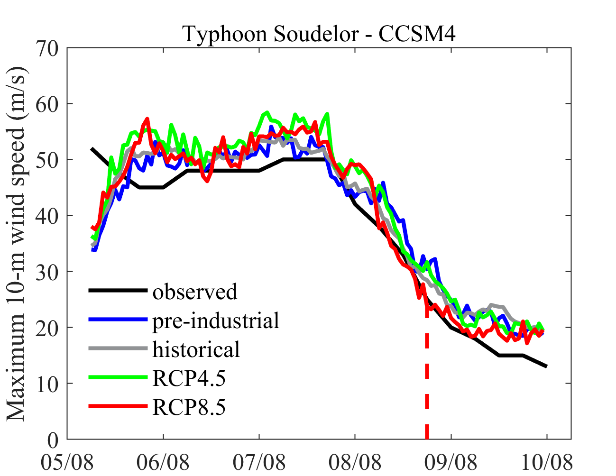

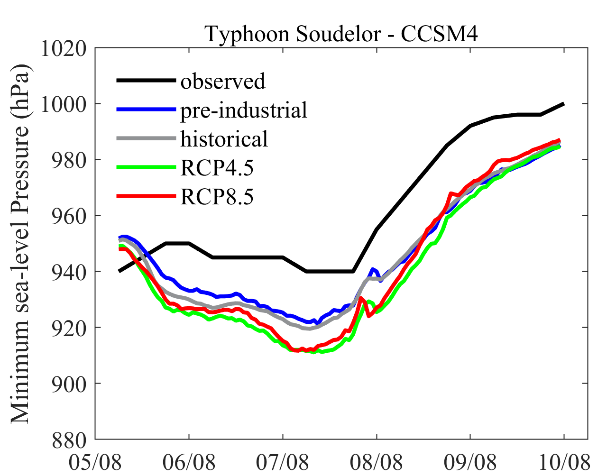


**(f)**

**(e)**

**Supplementary Figure 10:** Time series of (**a**, **c**, **e**) maximum 10-m wind speed and (**b**, **d**, **f**) minimum sea-level central pressure of Typhoon Soudelor. Historical simulation (2000-2020), pre-industrial simulation (1860-1880) and future typhoon simulation (2080-2100) under RCP 4.5 and RCP8.5 emission scenarios corresponding to FGOALS-s2 (**c**, **d**) and CCSM4 model (**e**, **f**). Observed landing time was marked with a vertical red dashed line in **a**, **c**, **e**.


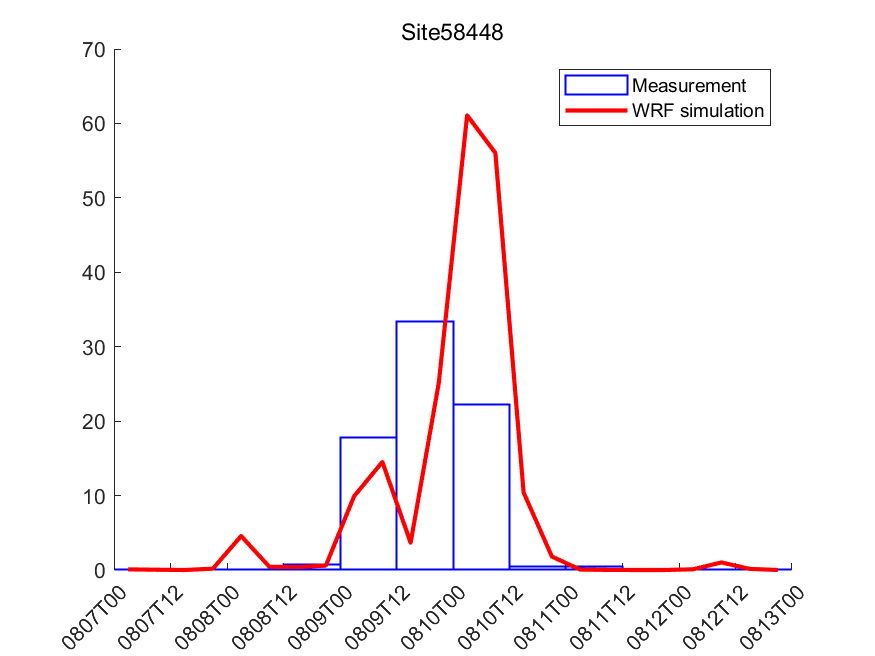

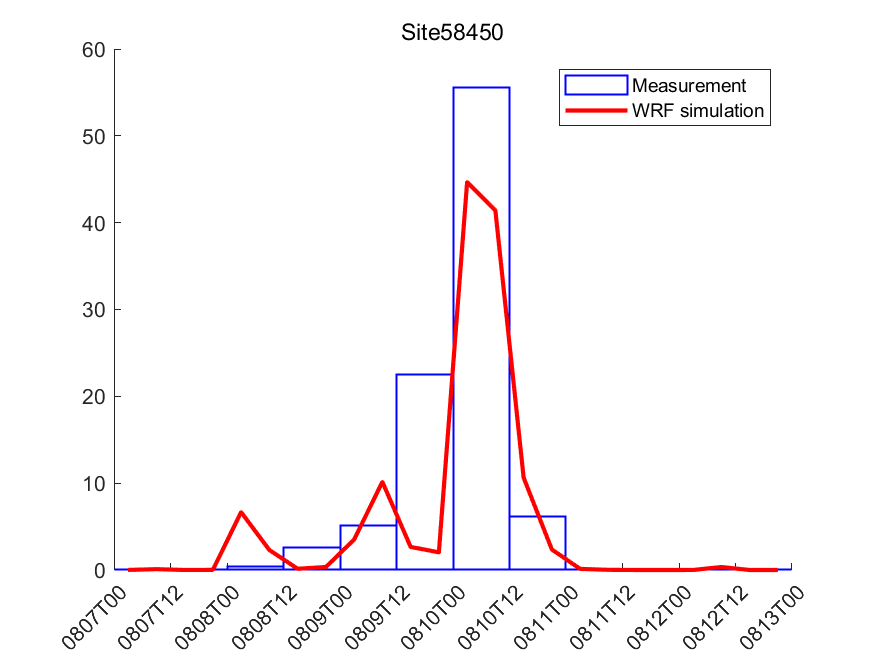


**(b)**

**(a)**


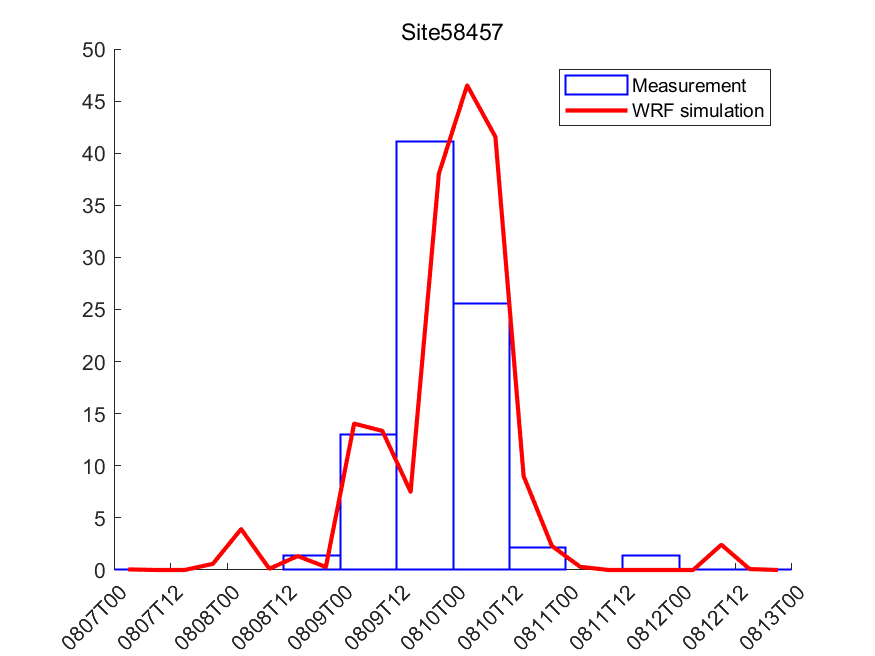

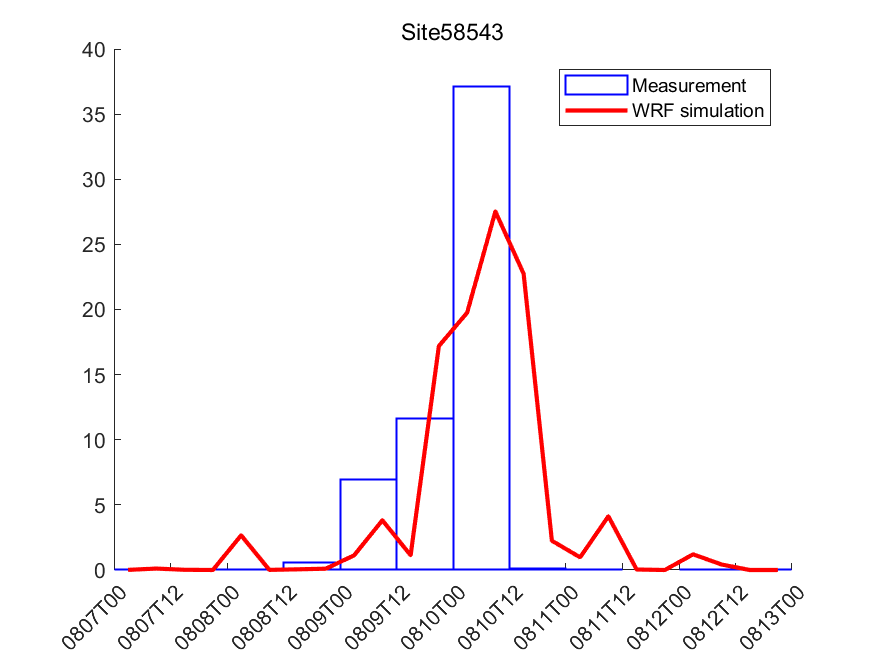


**(d)**

**(c)**


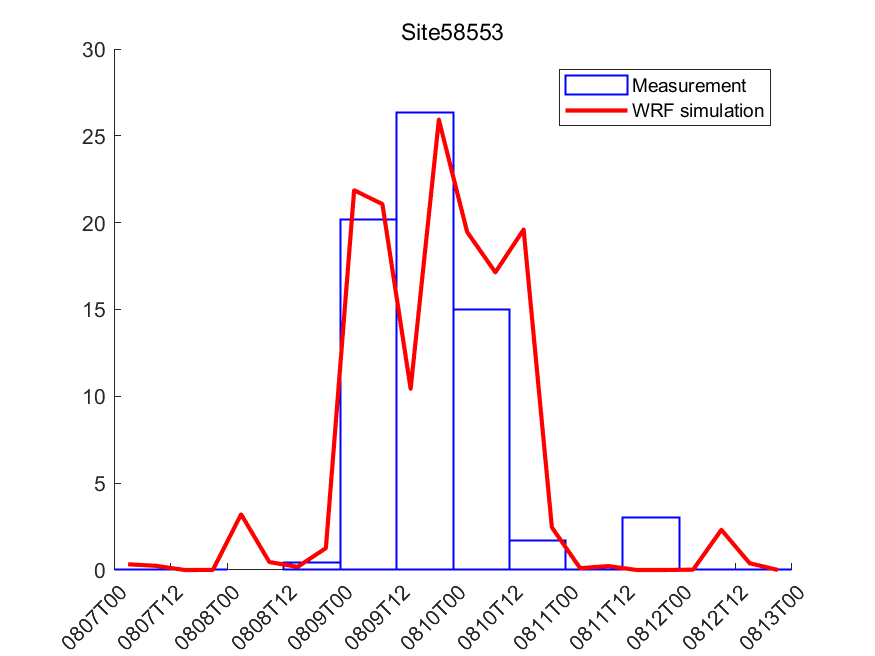

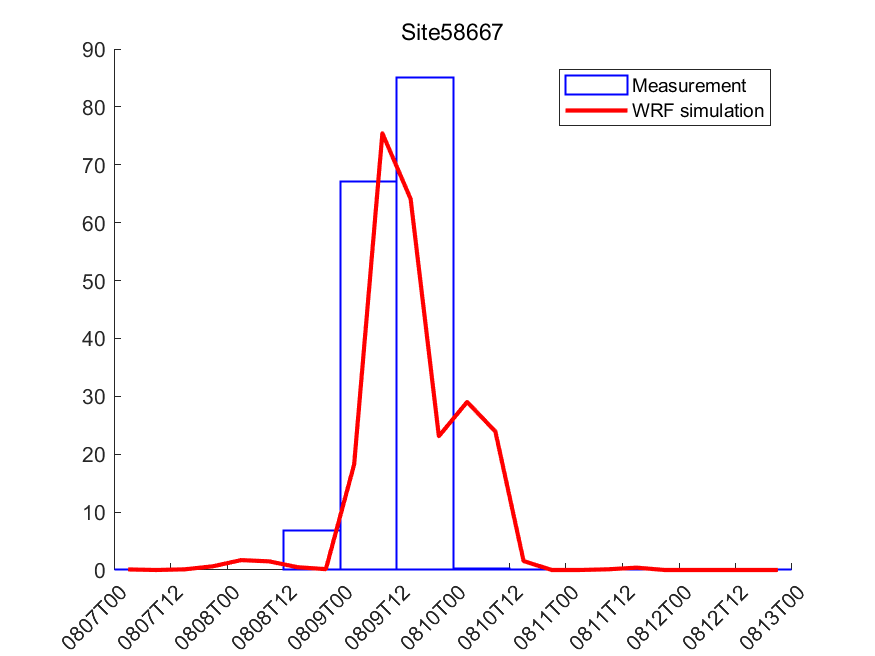


**(f)**

**(e)**

**Supplementary Figure 11:** Comparison of simulated 6-hour precipitation time series to the ground observation sites for Typhoon Lekima.


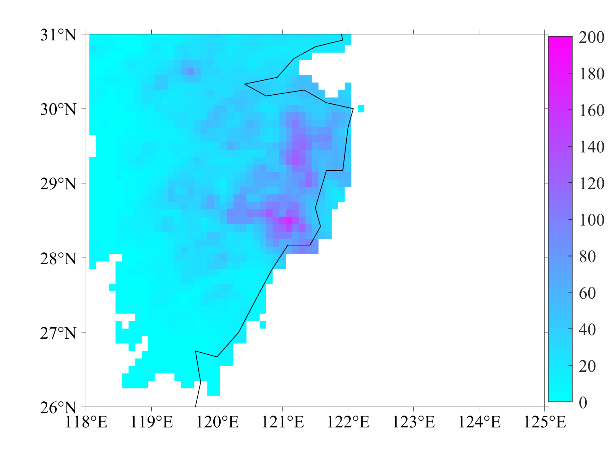

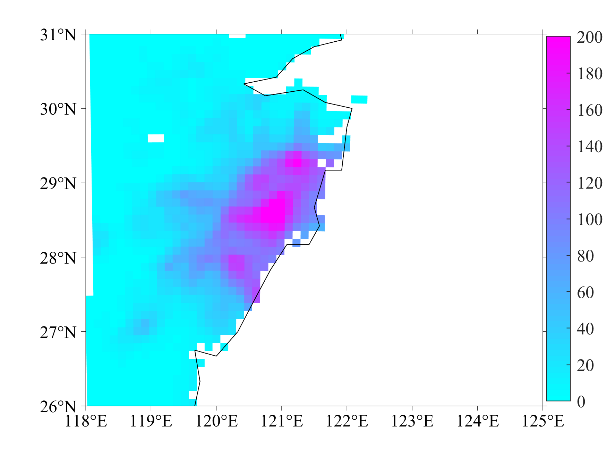


**(b)**

**(a)**


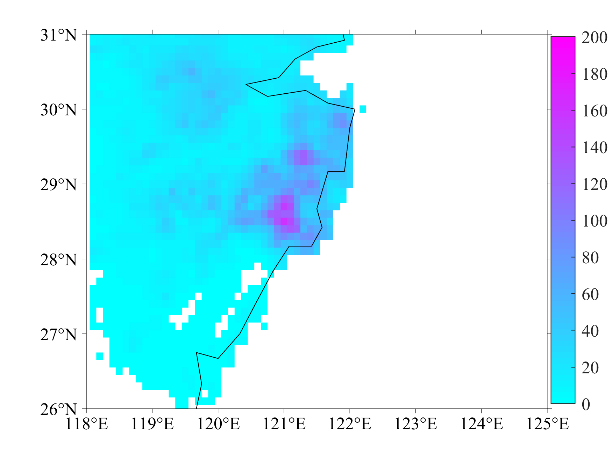

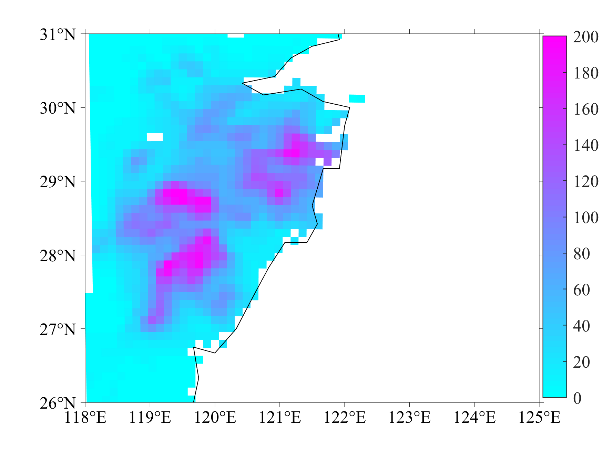


**(d)**

**(c)**

**Supplementary Figure 12:** Comparison of contours of 6-hour precipitation for Typhoon Lekima between CMA observation (**a**, **c**) and the WRF simulation (**b**, **d**). (**a**-**b**), during 2019-08-09 12 UTC to 2019-08-09 18 UTC. (**c**-**d**), during 2019-08-09 18 UTC to 2019-08-10 00 UTC. Maps were generated by MATLAB R2021a (http://www.mathworks.com/).


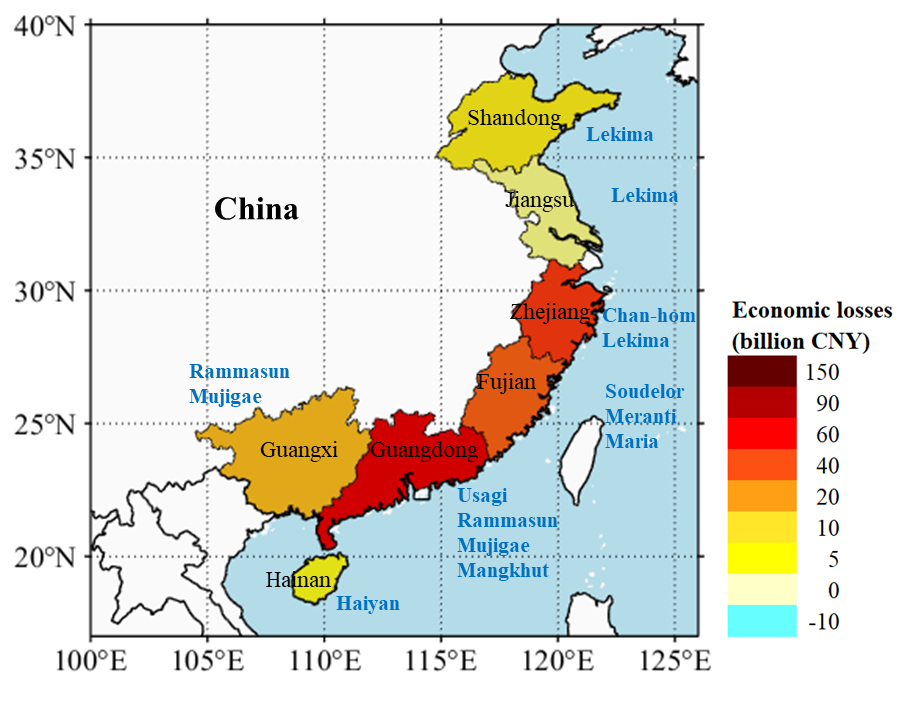


**Supplementary Figure 13:** Observed direct economic loss (CPI-2013 adjusted losses) in seven provinces of the 10 SuperTYs. Maps were generated by MATLAB R2021a (http://www.mathworks.com/).

**Supplementary Figure 14:** Comparison of historical observed direct economic losses (CPI-2013 adjusted losses), and DNN estimated losses from historical and future typhoon simulation for seven coastal provinces.

**
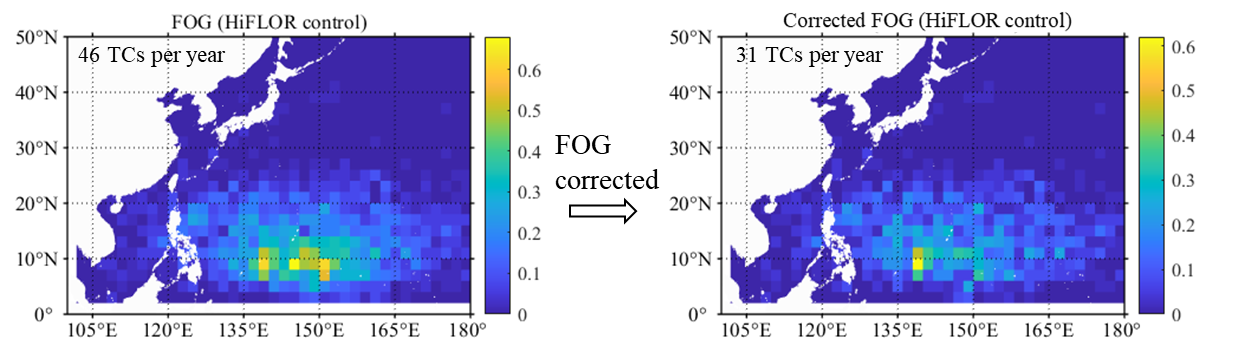
**

**(a)**

**
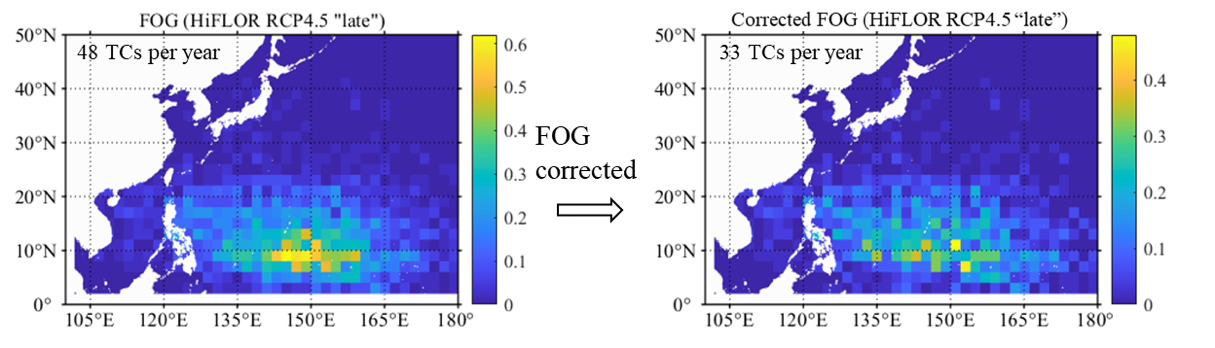
**

**(b)**

**Supplementary Figure 15:** Correction of FOG distribution in WNP from the HiFLOR control experiment (**a**), and the HiFLOR RCP4.5 “late” experiment (**b**). Maps were generated by MATLAB R2021a (http://www.mathworks.com/).

**(b)**

**(a)**

**(d)**

**(c)**

**Supplementary Figure 16:** The empirical CDFs of maximum 10-m wind speed series from the HiFLOR “late” experiment under RCP4.5, HiFLOR control experiment, the CMA-STI best track dataset and the corrected HiFLOR “late” projection for Zhejiang province (**a**), Fujian province (**b**), Guangdong province (**c**) and Hainan province (**d**).

**(b)**

**(a)**

**Supplementary Figure 17:** (**a**) Boxplots of economic loss index (ELI) predicted by the ensemble modelling for historical typhoons of Lekima, Soudelor, Usagi and Haiyan. The centre line denotes the median, the little solid square denotes the mean, box limits denote the 25th and 75th percentiles and whiskers are determined by the 5th and 95th percentiles. The observed ELI is marked with horizontal black lines. (**b**) The linear regression of targets (i.e., ELI Observations) relative to outputs (i.e., ELI predictions) for 20 testing typhoon samples. ELIs of 20 test samples were predicted by the ensemble-mean modelling.

**(b)**

**(a)**

**(d)**

**(c)**

**(f)**

**(e)**

**(h)**

**(g)**

**(j)**

**(i)**

**Supplementary Figure 18:** Predictions of ELI for ten SuperTYs under three future climate conditions. Usagi (**a**), Haiyan (**b**), Rammasun (**c**), Chan-hom (**d**), Soudelor (**e**), Mujigae (**f**), Meranti (**g**), Maria (**h**), Mangkut (**i**), Lekima (**j**). The centre line denotes the median, the little square denotes the mean, box limits denote the 25th and 75th percentiles and whiskers are determined by the 5th and 95th percentiles. The mean ELI corresponding to historical typhoon simulation is marked with a horizontal dashed black line.
